# Recent Progress of Anti-Freezing, Anti-Drying, and Anti-Swelling Conductive Hydrogels and Their Applications

**DOI:** 10.3390/polym16070971

**Published:** 2024-04-02

**Authors:** Ying Li, Qiwei Cheng, Zexing Deng, Tao Zhang, Man Luo, Xiaoxiao Huang, Yuheng Wang, Wen Wang, Xin Zhao

**Affiliations:** 1 College of Materials Science and Engineering, Xi’an University of Science and Technology, Xi’an 710054, China; 2 Department of Radiology, Functional and Molecular Imaging Key Lab of Shaanxi Province, Tangdu Hospital, Air Force Medical University, Xi’an 710038, China; 3 State Key Laboratory for Mechanical Behavior of Materials, Frontier Institute of Science and Technology, Xi’an Jiaotong University, Xi’an 710049, China

**Keywords:** conductive hydrogels, anti-freezing, anti-drying, anti-swelling, applications

## Abstract

Hydrogels are soft–wet materials with a hydrophilic three-dimensional network structure offering controllable stretchability, conductivity, and biocompatibility. However, traditional conductive hydrogels only operate in mild environments and exhibit poor environmental tolerance due to their high water content and hydrophilic network, which result in undesirable swelling, susceptibility to freezing at sub-zero temperatures, and structural dehydration through evaporation. The application range of conductive hydrogels is significantly restricted by these limitations. Therefore, developing environmentally tolerant conductive hydrogels (ETCHs) is crucial to increasing the application scope of these materials. In this review, we summarize recent strategies for designing multifunctional conductive hydrogels that possess anti-freezing, anti-drying, and anti-swelling properties. Furthermore, we briefly introduce some of the applications of ETCHs, including wearable sensors, bioelectrodes, soft robots, and wound dressings. The current development status of different types of ETCHs and their limitations are analyzed to further discuss future research directions and development prospects.

## 1. Introduction

Flexible conductive materials can convert external stimuli, such as stress, strain, temperature, and humidity, into electrical signals (such as current, resistance, and capacitance) [1,2,3]. Due to their unique stimuli-responsive characteristics, flexible conductive materials are commonly used in research fields such as human motion monitoring [4], human health monitoring [5], remote medical control [6], artificial skin [7], and soft robotics [8]. Traditional flexible conductive materials usually consist of flexible substrate materials (PDMS, polyurethane, polyetherimide, and Ecoflex) and rigid conductive materials (such as metal nanomaterials, carbon-based materials, and conductive polymers). The former provides stretchability, while the latter provides conductivity. However, traditional flexible conductive materials face challenges such as complex manufacturing processes, insufficient mechanical stretchability, poor compatibility with human tissues, and insufficient cohesion between the rigid conductive elements and the flexible substrate [9,10]. The limitations of traditional flexible conductive materials significantly impede large-scale commercial production and long-term utilization. Hence, it is of great research significance to develop flexible conductive materials with good biocompatibility, simple processing methods, excellent mechanical properties, high sensitivity, and long-term stable service.

In response to the aforementioned limitations, researchers have made significant strides in developing flexible conductive materials with hydrogels as the primary components. By controlling the composition and structure of hydrogels and integrating them with conductive materials, such as ion- or electron-conductive materials [11,12], materials with an excellent combination of mechanical properties (such as stretchability, compressibility, and fatigue resistance) and conductivity can be obtained [13]. Hydrogel-based flexible conductive materials exhibit mechanical properties similar to those of the skin, displaying good biocompatibility and stable and sensitive responses to external stimulus signals [14,15]. These qualities have greatly increased their application potential in the realm of sensing [16,17,18], including the monitoring of electrocardiogram and electroencephalogram signals [19], soft robotics [20], human motion monitoring [21], controlled drug release [22], photothermal therapy [23], and smart wound dressing [24,25].

Nevertheless, there are still outstanding issues in the actual application environment in research on conductive hydrogels. Due to the presence of a large amount of water and the hydrophilic network structure of their matrix, hydrogels have poor anti-freezing and anti-dehydration properties and insufficient swelling resistance. Consequently, their structure and functionality are compromised, severely constraining their applicability in complex environments. For instance, conductive hydrogels need to maintain their properties within a specific temperature range; indeed, when exposed to high or sub-zero temperatures, they become rigid and fragile due to water evaporation or water freezing, respectively. In addition, prolonged exposure to dry environments and water evaporation from the matrix affect their elasticity and conductivity [26]. Furthermore, conventional conductive hydrogels may exhibit substantial volumetric expansion due to the absorption of significant quantities of water from humid surroundings, reducing their mechanical and conductive properties [27]. This limits their applications under extreme conditions [28,29,30]; therefore, the development of hydrogels with environmental tolerance is imperative to broadening their application scope in flexible sensing and biomedical areas.

Recently, researchers have devised a range of strategies aimed at tackling these obstacles, such as adding anti-freezing organic solvents, using salt solutions, and performing surface modification with hydrophobic elastomers to reduce water evaporation and swelling or prevent conductive hydrogels from freezing [31,32,33]. Additionally, some methods also involve the modification of the network structure with chemical or physical binding to promote the anti-swelling performance of conductive hydrogels [34]. With these methods, environmentally tolerant conductive hydrogels (ETCHs), which possess improved anti-freezing, anti-drying, and anti-swelling properties, have been successfully created. Such advancements allow for the effective use of conductive hydrogels in flexible sensors, bionic soft robots, biomedical materials, and other fields under extreme environments while maintaining their excellent stability and durability.

**Scheme 1:**
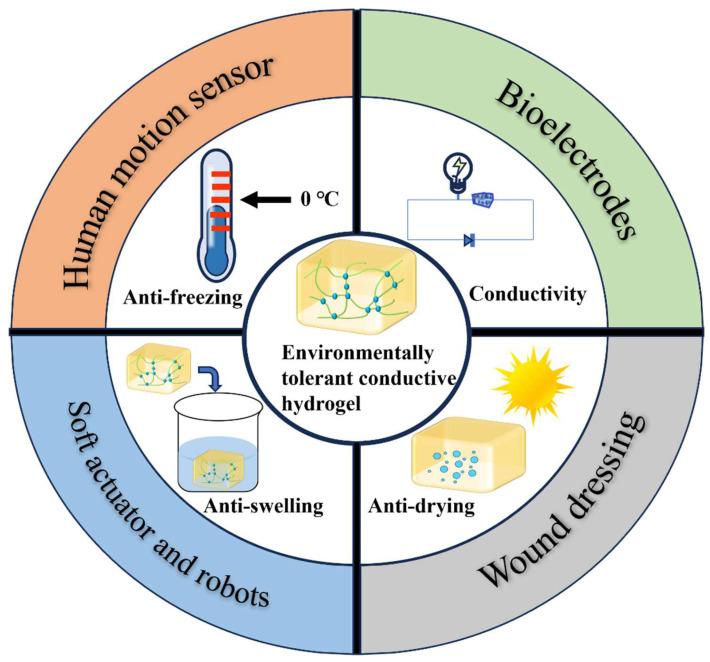
Schematic of the functionalized ETCHs and their applications.

In this study, as shown in Scheme 1, we reviewed recent works on ETCHs with anti-freezing, anti-drying, and anti-swelling properties, providing a brief overview of the current design strategies employed for their development. Additionally, we explored potential applications based on the distinctive attributes of ETCHs, concluding that these multifunctional anti-freezing, anti-drying, and anti-swelling conductive hydrogels have great potential for future development.

## 2. Structure Design and ETCH Preparation Methods

### 2.1. ETCHs with Anti-Freezing and Anti-Drying Characteristics

#### 2.1.1. Immersion in Salt Solution 

**Figure 1:**
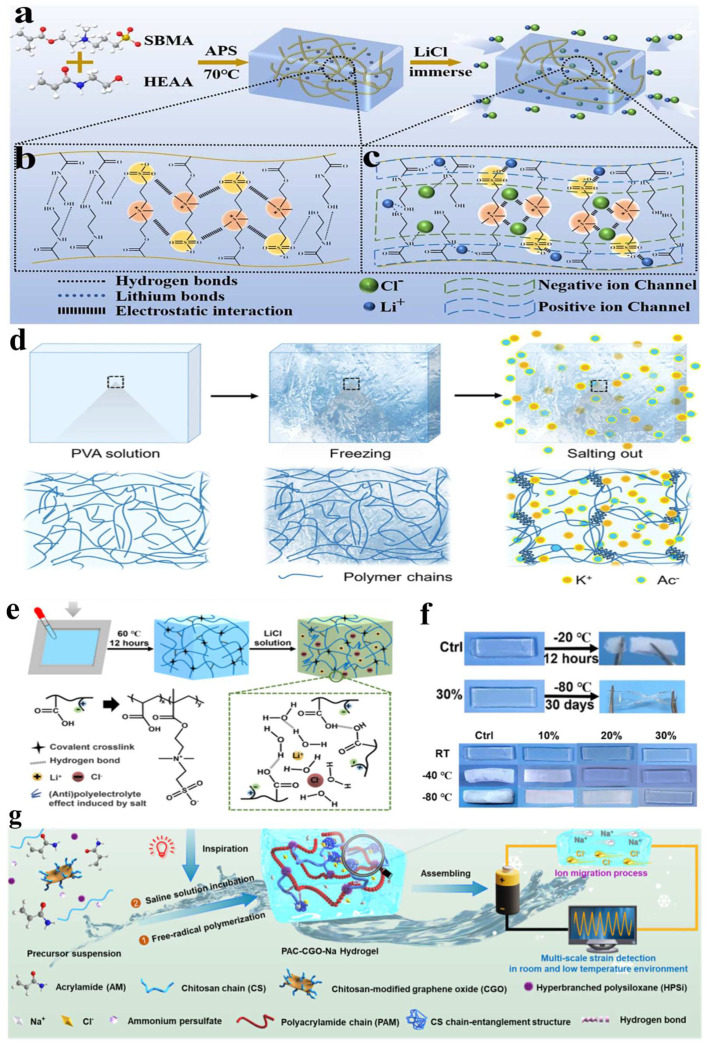
(**a**) Preparation process of SBMA-HEAA hydrogel. (**b**) Interactions among the anti-freezing hydrogel. (**c**) Ion-transmission mechanism of anti-freezing hydrogel. From ref. [38] used with permission by Royal Society of Chemistry. (**d**) Preparation of hydrogel with PVA and KAc. From ref. [39] used with permission by Royal Society of Chemistry. (**e**) Monolithic structure of environmental ionic hydrogel. (**f**) Anti-freezing property of LiCl solution and ionic hydrogel. From ref. [40] used with permission by Elsevier. (**g**) Schematic diagram of anti-freezing nanocomposite conductive hydrogel. From ref. [41] used with permission by Elsevier.

Traditional conductive hydrogels are prone to freezing when the internal water reaches the freezing point, leading to flexibility and functionality loss. To increase the operational temperature range for the utilization of conductive hydrogels, as demonstrated in Table 1, researchers have found that supplementing them with inorganic salt solutions (LiCl, NaCl, CaCl_2_, etc.) can be an effective anti-freezing and anti-drying strategy [35,36,37], as doing so lowers the freezing point, reducing the evaporation of water in the hydrogel network. The underlying mechanism consists of the salt solution disrupting the arrangement of the water molecules and increasing the intermolecular forces among them. The dissolved salt is dissociated into ions; as a result, ions interact with water to form hydrated ions, making it difficult for water molecules to form a regular crystalline structure and less likely for water to evaporate. Additionally, a salt solution can also provide good ion conductivity to hydrogels. For instance, Zhang et al. [38] utilized zwitterionic sulfobetaine methacrylate (SBMA) and N-(2-hydroxyethyl) acrylamide (HEAA) for copolymerization at 70 °C. Then, the resulting hydrogel was immersed in a 6 M LiCl solution for soaking to create an anti-freezing and anti-drying ion-conductive hydrogel. The addition of LiCl not only endowed the hydrogel with high ion conductivity (25.8 S/m) at room temperature but also allowed it to maintain good conductivity (2.21 S/m) even at a low temperature (−40 °C), as demonstrated in Figure 1a–c. In addition, the incorporation of a salt solution might improve the mechanical performance of hydrogels. An example of this technique is given in the work by Wu et al. [39], who successfully balanced the anti-freezing and mechanical properties of hydrogels and advanced their application in sub-zero-temperature environments. As shown in Figure 1d, the authors used a salt solution replacement strategy to prepare high-toughness and anti-freezing ion-conductive hydrogels by freezing polyvinyl alcohol (PVA) hydrogel precursor solution and immersing it in potassium acetate (KAc) solution for 96 h. Due to the salting-out effect on PVA by KAc, the hydrogel achieved the best anti-freezing performance when the salt concentration was adjusted to 50 wt%. The resultant hydrogel could withstand a low temperature (−60 °C) while simultaneously possessing a conductivity value of 1.2 S/m. Moreover, the salting effect on PVA by KAc significantly enhanced the mechanical properties of the hydrogel, which demonstrated an ultimate tensile strength of 8.2 MPa and toughness of 25.8 MJ/m^3^. Another example is displayed in Figure 1e; Zhang’s group [40] developed a poly(SBMA-co-AA) hydrogel based on zwitterionic sulfobetaine methacrylate (SBMA) and charged monomer acrylic acid (AA) and then immersed it in LiCl solutions with various weight percentages to modify its mechanical, anti-freezing, and anti-drying properties, as well as its ionic conductivity. As shown in Figure 1f, the addition of LiCl effectively lowered the freezing point of the hydrogel (−9.25 °C kg mol^−1^), which displayed good transparency for 30 days even at −80 °C after having been absorbed in a 30 wt% LiCl solution. In addition, the anti-freezing performance of the hydrogel could be enhanced with the increase in the concentration of LiCl solution. Moreover, the hydrogel sample stored at 25 °C and 54% humidity for up to one week retained nearly 100% of its water content. Interestingly, lyophilized conductive hydrogels can spontaneously absorb water for recyclable use, which extends their service career, an essential characteristic for their application in extreme environments. Wu et al. [41] developed a conductive hydrogel consisting of polyacrylamide (PAM), chitosan (CS), and chitosan-modified graphene oxide nanosheets (CGO nanosheets). Then, the hydrogel was immersed in NaCl salt solution to yield the final anti-freezing conductive hydrogel. The high-concentration NaCl salt solution (21.2 wt%) significantly changed the network structure of the hydrogel by allowing water molecules to form hydrogen bonds (Figure 1g), resulting in exceptional mechanical and anti-freezing properties. Even when exposed to the cold temperature of −20 °C for 30 days, the hydrogel preserved satisfactory conductivity (4.10 S/m). With this breakthrough study, the authors paved the way for the future development of ion-conductive hydrogels for application in extreme environments.

In summary, the incorporation of salt solutions can greatly improve the anti-freezing, anti-drying, and ionic conductivity properties of conductive hydrogels. However, this preparation method might have a negative impact on the equilibrium between the mechanical properties and electrical conductivity of hydrogels. In addition, some salt solutions might be toxic to humans and prevent the utilization of conductive hydrogels in the biomedical field. Therefore, the selection of an appropriate salt solution remains a crucial aspect in designing anti-freezing and anti-drying conductive hydrogels with desirable mechanical and biocompatible performance.

#### 2.1.2. Incorporation of Organic Solvent 

**Figure 2:**
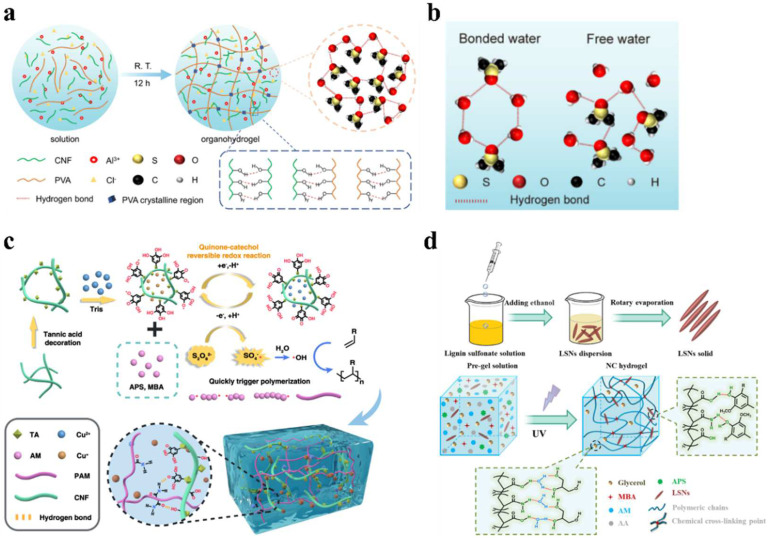
(**a**) The synthesis process of PVA-CNF organohydrogel. (**b**) Schematic diagram of the hydrogen bonds. From ref. [46] used with permission by Elsevier. (**c**) The preparation process of Cu-TA/CNF organohydrogel. From ref. [47], used with permission by Elsevier. (**d**) The synthesis process of an anti-freezing hydrogel and hydrogen bond within hydrogel. From ref. [48], used with permission by Elsevier.

Directly incorporating organic solvents with inherent anti-freezing properties before the crosslinking of conductive hydrogels is another method for improving the freezing resistance of these materials [42]. Researchers have reported that the freezing point of the liquid phase in conductive hydrogels can be successfully reduced with the incorporation of organic solvents with a freezing point lower than that of water. As shown in Table 2, this innovative approach has facilitated the development of double-solvent system-based conductive hydrogels with anti-freezing and anti-drying characteristics [43,44,45]. As a representative example, Song et al. [46] utilized a binary solvent composed of dimethyl sulfoxide (DMSO/water 1:2) as a dispersion medium to dissolve polyvinyl alcohol (PVA), cellulose nanofibers (CNFs), and aluminum chloride hexahydrate (AlCl_3_·6H_2_O), fabricating an ion-conductive hydrogel with anti-freezing and anti-drying properties (Figure 2a). The nanocomposite organohydrogel prepared by using a simple one-pot method exhibited high tensile strain (up to 696%), high toughness (3.54 MJ/m^3^), excellent moisture retention performance (85% hydrogel weight retained after 30 days), and a wide working temperature range (from −50 °C to 50 °C). Furthermore, the PVA-CNF organohydrogel exhibited remarkable flexibility, stretchability, and compressibility even at the extremely low temperature of −50 °C. This unique behavior can be attributed to the formation of strong hydrogen bonds between DMSO and water (Figure 2b), allowing the hydrogel to regain its original shape after compression. The results also demonstrate the significant role of DMSO in lowering the freezing point of water, resulting in an organohydrogel exhibiting remarkable resistance to freezing and drying. Notably, after 30 days of storage at room temperature, the organohydrogel system lost only 15% of its initial mass. In addition to DMSO, glycerol is another organic solvent widely used for producing anti-freezing and anti-drying conductive hydrogels. Jiang’s team [47] took inspiration from mussels to develop a time-efficient method for creating multifunctional hydrogels with adhesion, anti-freezing, and anti-drying properties. As shown in Figure 2c, the hydrogel was produced by employing tannic acid-modified cellulose fibers (TA@CNFs), PAM, metal Cu^2+^, and glycerol. By utilizing the glycerol–water (50 wt%) binary solvent in the presence of Cu^2+^ and the TA@CNF catalytic system, the polymer was rapidly polymerized to form a network structure, a method which was found to be much faster than the traditional thermally induced free-radical polymerization method. Further, this process contributed to the hydrogen bond interaction between glycerol and water molecules, effectively suppressing the freezing of water; as a result, the conductive hydrogel exhibited long-term stability in extremely cold environments. Zhang et al. [48] developed UV-blocking nanoparticle-lignin sulfonate nanorods (LSNs) and incorporated them into a polyacrylamide/polyacrylic acid (PAM/PAA) polymer network by using a simple method. Firstly, dispersed lignin sulfonate solution was supplemented with anhydrous ethanol and then subjected to rotary evaporation to form spindle-like nanorods. Then, the LSNs and AM/AA were dissolved in a water/glycerol (6:1) binary solution to obtain an anti-freezing, anti-drying, and UV-shielding hydrogel (Figure 2d). Due to the LSNs and hydrogen bonding between water and glycerol, the obtained hydrogel could withstand the extremely cold temperature of −60 °C for a week and maintain the initial state after storage at room temperature (60% relative humidity) for 15 days. Gao et al. [49] developed a chitosan–gelatin–glycerin–NaCl (CGGN) organohydrogel film sensor with a thickness of only 0.1 mm, which is sensitive to humidity and temperature. Due to the binary solvent composed of water/glycerin, this sensor possesses a wide relative humidity (RH) range (20–90%), high transparency, and anti-freezing properties (−30 °C~50 °C). The above work provides a basis to introduce novel methodologies for fabricating a new generation of electronic skin sensors and wearable devices capable of discerning multiple stimuli.

**Figure 3:**
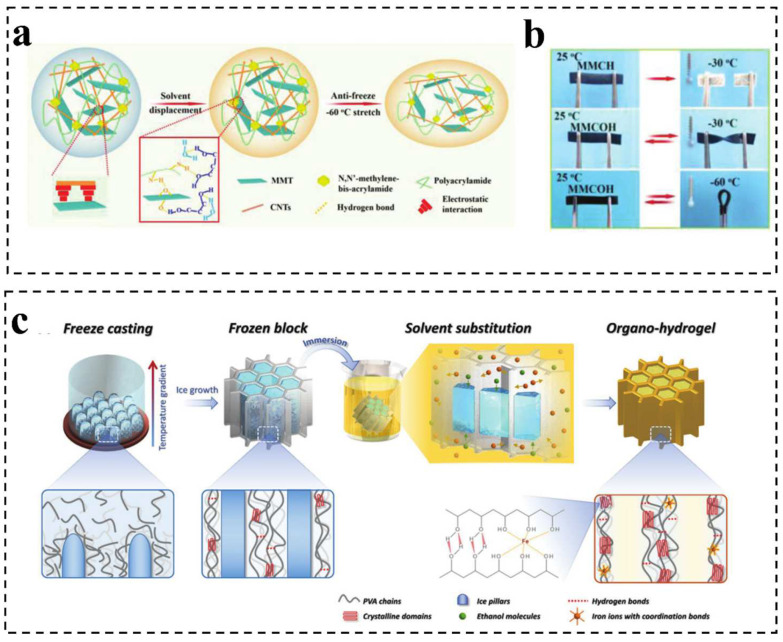
(**a**) The preparation process of anti-freezing conductive hydrogel. From ref. [55] used with permission by John Wiley and Sons. (**b**) Images of anti-freezing property of conductive hydrogel. From ref. [55], used with permission by John Wiley and Sons. (**c**) The freeze casting assisted with solution substitution approach to prepare PVA-based conductive organohydrogel. From ref. [56], used with permission by John Wiley and Sons.

Besides directly incorporating organic solvents before the crosslinking of hydrogels, another strategy that has also been effectively employed in engineering anti-freezing and anti-drying conductive hydrogels is organic solvent substitution, whereby some of the water molecules within the internal hydrogel network are replaced with organic solvents through molecular diffusion, endowing these materials with anti-freezing and anti-drying characteristics [50,51]. Many researchers have adopted this approach to improve ETCHs [52,53,54]. For example, Sun et al. [55] prepared a high-toughness ETCH composed of PAM, montmorillonite (MMT), and carbon nanotubes (CNTs) and subjected it to glycerol solvent substitution (Figure 3a). As shown in Figure 3b, the obtained organohydrogel exhibited a wide working temperature range (from −60 °C to 60 °C) and maintained excellent conductivity, as well as remarkable stretchability, at −60 °C for up to 30 days. The hydrogen bonding between water and glycerol molecules endowed the organohydrogel with exceptional anti-freezing properties. This strategy effectively addresses the challenges faced by conductive hydrogels. In another work, Zhai et al. [56] designed a robust and freezing-resistant conductive hydrogel by using freeze casting and solution substitution (Figure 3c).

**Table 2:** Performances of anti-freezing and anti-drying conductive hydrogel by incorporating organic solvents. “-” means not investigated.

Materials	Solvent Composition	Anti-Freezing Temperature	Anti-Drying Property	Application	Ref.
PVA/SNF/CN	Ethylene glycol/water	−18 °C	-	Human motion detection	[42]
PVA/EG	Ethylene glycol/water	−20 °C	-	Human motion monitoring	[43]
(PVA)/CNCs-PDDA/PA	Glycerol/water	−30 °C	-	Strain sensors	[45]
PVA/CNF	DMSO/water (1:2 molar ratio)	−50 °C	85% of hydrogel weight retention after 30 days	Human motion sensor	[46]
AM/Cu-TA/CNF	Glycerol/water (50 wt%)	−20 °C	70% weight retention after 7 days	Strain sensor	[47]
PAM/PAA/LSNs	Glycerol/water (6:1)	−60 °C	Keep the initial state for 7 days	Energy storage	[48]
PAM/MMT/CNTs	Glycerol/water (6:1)	−60 °C	90% of hydrogel weight retention after 30 days	Human motion sensor	[55]
PVA	Ethanolic ferric chloride (2 wt%)	−30 °C	-	Strain sensor	[56]
Cu-TA/CNF/G	50 wt% EG	−30 °C	90% of hydrogel weight retention after 60 days	Human motion sensor	[57]

The PVA solution was first unidirectionally frozen by using liquid nitrogen and subsequently immersed in a pre-cooled ethanolic ferric chloride (2 wt%) replacement solution for three days. Following the vertical gradient decrease in temperature, the PVA polymer chains unidirectionally crystallized and aggregated; additionally, the coordination interaction between Fe^3+^ and the hydroxyl groups of the PVA polymer chains made the latter further aggregate during the solution substitution process. Thence, the prepared conductive PVA hydrogel was found to have high tensile strength (6.5 MPa), excellent tensile strain (1710%), and good electrical conductivity (6.5 S/m). Furthermore, the ethanol substitution solvent imparts anti-freezing properties to organohydrogels, enabling their utilization in extreme environments. In addition to these organic solvents, ethylene glycol can also be used as an anti-freezing and anti-drying replacement solvent. For example, Han et al. [57] developed an anti-freezing, anti-drying, and conductive organohydrogel based on PAM, cellulose nanocrystals modified with tannic acid (CNC-TA), and graphene (G). The free water in the hydrogel network was thereafter replaced with ethylene glycol (50 wt%) to prepare the target ETCH.

The final ETCH displayed outstanding freezing resistance (ability to withstand a temperature of −30 °C for 24 h) and impressive moisturizing properties (ability to withstand a temperature of 60 °C for 96 h) due to the interactions between water and ethylene glycol. 

After adding organic solvents to a hydrogel system, the free water molecules in the hydrogel network form hydrogen bonds with the organic solvent molecules, reducing the transition temperature of the liquid phase within the hydrogel, which is beneficial for inhibiting the formation of ice crystals and preparing conductive hydrogels with anti-freezing and anti-dehydration properties. However, the incorporation of an organic solvent also presents some limitations and problems. For example, compared with the salt solution system mentioned in the previous section, organic solvents are prone to oxidation or decomposition, which would affect the electrical conductivity of the final hydrogel. In addition, in application scenarios such as human motion sensing and wound dressing, some organic solvents may reduce the biocompatibility of hydrogels, limiting their use in humans. Therefore, the selection of organic solvents needs to be comprehensively considered in view of different application scenarios.

#### 2.1.3. Surface Modification 

In nature, plants and animals possess the ability to store a significant amount of water within their bodies. Moreover, they can encapsulate and immobilize water molecules through their unique skin structures. Taking inspiration from this natural mechanism, researchers have surface-modified conductive hydrogels by incorporating an elastomer or hydrophobic layer as a barrier to prevent water loss and freezing [58,59]. As demonstrated in Table 3, incorporating an elastomer or hydrophobic layer can suppress the water molecule diffusion process, creating a diffusion barrier for the conductive hydrogel and isolating it from the surrounding environment. This design concept can be likened to the conductive hydrogel wearing “protective clothing” that shields it from external factors [60,61].

**Figure 4:**
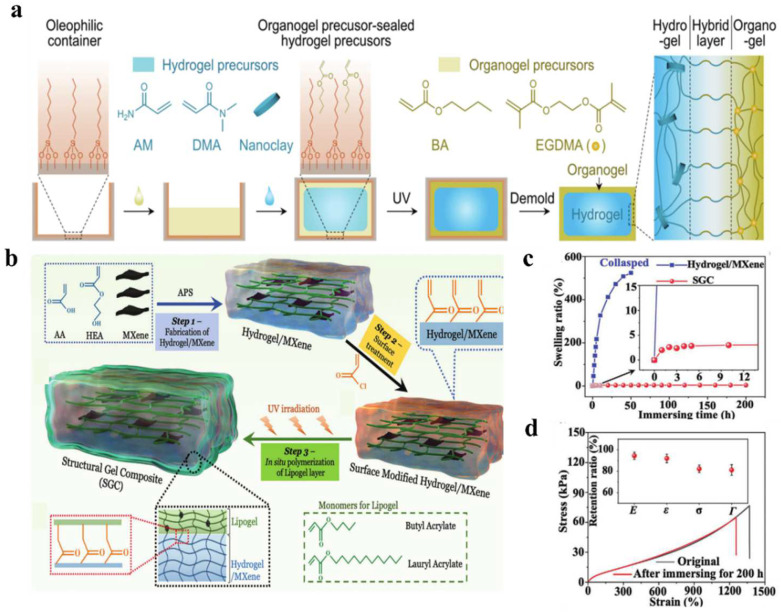
(**a**) The bioinspired water retention hydrogel based on hydrophobic coating. From ref. [62], used with permission by John Wiley and Sons. (**b**) The fabrication of hydrophobic lipogel/MXene conductive hydrogel. (**c**) The anti-swelling property of hydrogel/MXene and SGC. (**d**) The stress–strain curves of hydrogel/MXene and SGC after immersion of 200 h. From ref. [63], used with permission by John Wiley and Sons.

Zhang et al. [62] prepared a simple yet effective wet immersion strategy to produce organically sealed dehydrated hydrogels with excellent water retention performance. The authors made use of the preferential spreading on hydrophilic–oleophilic surfaces of the liquid precursor solution and its fluidity to create a wetting-induced organic gel precursor layer, completely sealing the hydrogel precursor. The thickness of this seal was controlled by adjusting the viscosity of the organic gel precursor solution. Subsequently, through a one-step interfacial polymerization process, a hydrogel sealed with an organic gel was obtained. In summary, this method involves the wetting-induced sealing of the hydrogel precursor with a three-dimensional hydrophobic–oleophilic organogel substrate to obtain an anti-drying and anti-swelling organohydrogel with a core–shell structure (Figure 4a). Compared with the addition processes, this effective design is simpler and allows for the construction of 3D hydrogels with various shapes. Ultimately, in the above study, the final organohydrogel possessed a weight retention rate of 95 wt% after 2 days and a swelling ratio of 3–9 wt% after 7 days. Wang et al. [63] developed a composite hydrogel structure by incorporating a lipid hydrogel layer with a conductive hydrogel. By polymerizing AA, 2-hydroxyethyl acrylate (HEA), and MXene nanosheets at room temperature, double bonds were introduced onto the hydrogel surface through the reaction between hydroxyl groups and acrylamide. Subsequently, a lipogel was created through in situ polymerization initiated by using ultraviolet light, resulting in a hydrophobic layer on the surface of the hydrogel (Figure 4b). As shown in Figure 4c,d, the final hydrogel had excellent anti-drying (about less than 10% weight loss after 200 h in an open-air environment) and anti-swelling properties (approximately 3% volume expansion in water after 200 h). Even after having been immersed in water for 200 h, the composite hydrogel maintained fracture strain (around 1350%) and tensile strength (approximately 75 kPa). The hydrophobic coating is compatible with the modulus of human skin, allowing the gel sensor to exhibit precise sensitivity and making it suitable for designing multifunctional wearable sensors for underwater applications.

Surface modification not only confers anti-drying properties to conductive hydrogels but also enhances their durability. However, this method also has some drawbacks. For instance, the modified layers tend to be insulating, and the lack of appropriate electrical conductivity regulation might be detrimental to conductive hydrogel applications in the sensing field. In addition, some elastic coating layers may experience weak adhesion and mismatching Young’s moduli at the interface with conductive hydrogels, which can lead to delamination and detachment between the modified layer and the hydrogel. Therefore, the stability and durability of the interface between the modified layer and the conductive hydrogel remains a significant issue and should be carefully considered in the design stage before modification.

#### 2.1.4. Other Strategies

**Figure 5:**
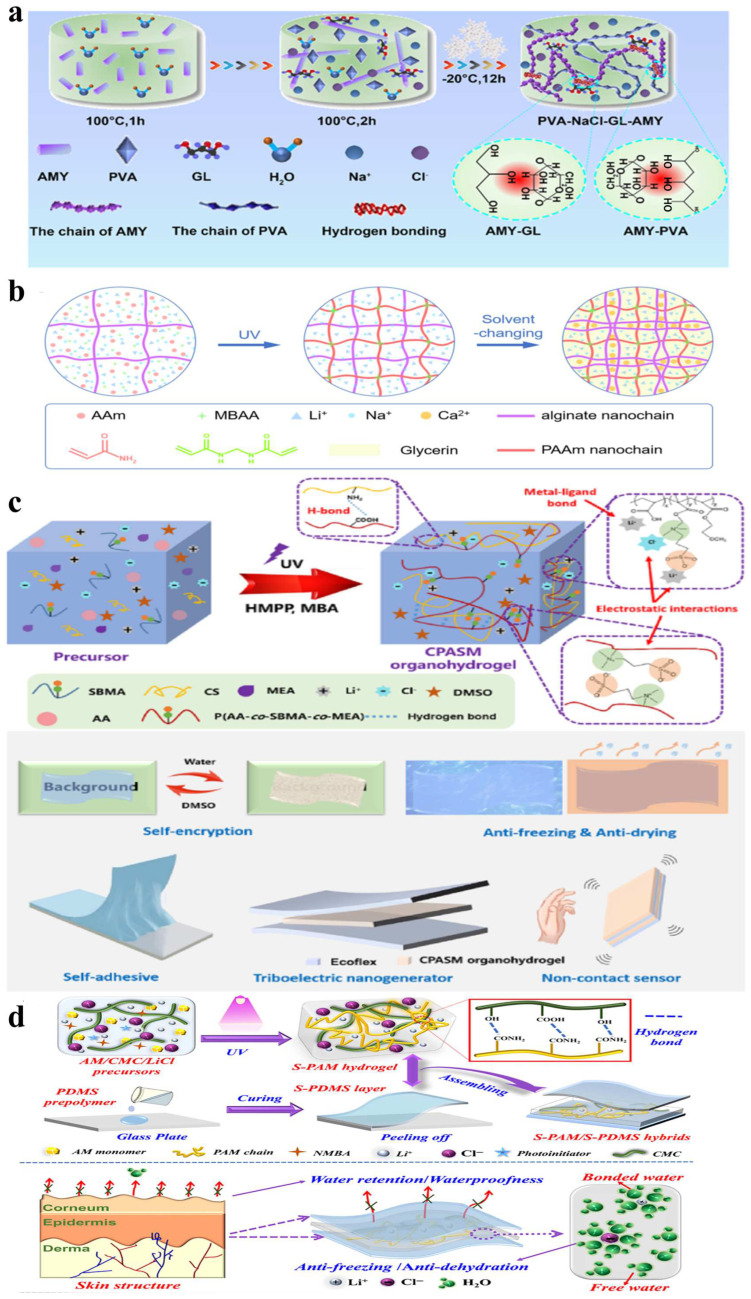
(**a**) The preparation scheme of PVA/AMY/NaCl conductive organohydrogel combined with NaCl and glycerol. From ref. [64], used with permission by Royal Society of Chemistry. (**b**) The fabrication process of conductive organohydrogel with solvent replacement with LiCl/CaCl_2_ and glycerol. From ref. [65], used with permission by Elsevier. (**c**) The fabrication process of CPASM organohydrogel assisted with Ecoflex sandwiching modification. From ref. [66], used with permission by Elsevier. (**d**) The preparation process of composite PAM/CMC hydrogel with PMDS sandwiching modification. From ref., [67] used with permission by John Wiley and Sons.

In addition to the aforementioned methods, some researchers have combined two or more methodologies to create ETCHs with freezing resistance, anti-drying characteristics, long-term stability, and tunable mechanical properties, as shown in Table 4. For example, Jia et al. [64] successfully prepared a conductive hydrogel with anti-freezing and anti-drying properties by integrating amylose into a PVA/glycerol/NaCl hydrogel with the freeze–thaw method (Figure 5a). Amylose possesses numerous hydroxyl groups that can interact with PVA and glycerol to enhance the toughness and moisturizing properties of hydrogels. In the experiments, after seven days, the hydrogel maintained a mass ratio close to 85%. Through a synergistic effect, glycerol and NaCl reduced the freezing point (−34.18 °C) of the aqueous phase within the hydrogel, allowing it to increase by up to 702% at −20 °C. Wang et al. [65] proposed a high-toughness, anti-freezing, and anti-drying organohydrogel by using sodium alginate (SA)/PAM and the organic solvent and salt solution replacement method (Figure 5b). The SA/PAM hydrogel was first fabricated; then, it was immersed in a LiCl/CaCl_2_/glycerol/water solution for water replacement in the hydrogel matrix. Compared with the ordinary hydrogel without glycerin, the prepared organohydrogel showed outstanding moisture retention for 30 days, great anti-freezing performance at the temperature of −80 °C, and better mechanical performance (the tensile stress was approximately 0.81 MPa). The above work proposed a novel and simple method to produce organohydrogels with anti-freezing and anti-drying properties. Due to their enhanced durability and stability, they show great potential for applications in electronic skin and soft wearable devices. Yuan et al. [66] fabricated a stretchable, anti-freezing, moisturizing, and ion-conductive multifunctional organohydrogel, named CPASM, composed of AA, SBMA, ethylene glycol methyl ether acrylate (MEA), CS, and a binary solvent system of DMSO/water (Figure 5c). The hydrogen bonding between the organic solvent and water decreased the freezing point of water. In turn, the organohydrogel was able to resist freezing even at −30 °C. Moreover, creating a sandwich structure with Ecoflex elastomer on the surface of the organohydrogel resulted in good anti-drying performance, with 80% of water being preserved at room temperature. Additionally, the zwitterion interactions and hydrogen bonds within the organohydrogel played a crucial role in improving its mechanical properties. Han et al. [67] developed an ETCH based on sodium carboxymethyl cellulose (CMC), PAM, and LiCl and modified it with a sandwich structure based on polydimethylsiloxane (PDMS) elastomer (Figure 5d). To prevent the PDMS elastomer layers from detaching themselves from the hydrogel, the surface of the hydrogel was precoated with silane to induce the formation of strong covalent bonding with PDMS.

The obtained hybrid hydrogel exhibited excellent anti-fatigue mechanical performance under 70% tensile strain for 100 stretching–recovery cycles, long-term moisture retention (more than 98% weight retention after 15 days), and a broad working temperature range (from −20 °C to 60 °C). Flexible sensors assembled by using this multifunctional hydrogel could receive signals and provide stable feedback in extreme environments. Wu et al. [68] prepared intrinsically stretchable, transparent, and high-performance thin-film humidity sensors by using PAM/PDMS/carrageenan hydrogel thin films. The thickness of the films was adjusted to 6.06 μm; then, they were immersed in a lithium bromide (LiBr) solution, which enhanced their anti-drying and anti-freezing properties. This high-performance, stretchable, and transparent thin-film sensor is considered to be a high-efficiency strategy for the future fabrication of wearable devices.

**Table 4:** Performances of anti-freezing and anti-drying conductive hydrogel by other strategies. “-” means not investigated.

Materials	Strategy	Solvent Composition	Anti-Freezing Temperature	Anti-Drying Property	Application	Ref.
PVA/AMY/NaCl	Organic solvent and salt solvent incorporation	Glycerol/NaCl/water	−20 °C	85% of hydrogel weight retention after 7 days	Human motion sensor	[64]
SA/PAM	Organic solvent and salt solvent replacement	LiCl/CaCl_2_/Glycerol/water	−80 °C	80% of hydrogel weight retention	Electronic skin	[65]
SBMA/AA/CS/MEA	Binary organic/water solvent and elastomer modification	DMSO/water	−30 °C	80% of hydrogel weight retention	Human motion sensor	[66]
PAM/CMC	Binary salt/water solvent and elastomer modification	LiCl/water	−20 °C	40% of hydrogel weight retention after 15 days	Human motion and strain monitor	[67]
AM/PVA/AFP	Nature AFP	Water	−15 °C	-	Human motion sensor	[69]

Inspired by nature, freeze-resistant components based on cold-temperature-tolerant organisms have also been incorporated into conductive hydrogels to enhance their anti-freezing properties. For example, Guo’s group [69] developed a modifiable anti-freezing hydrogel by adding natural fish-derived anti-freezing proteins to the hydrogel system. The hydrogel consisted of chemically crosslinked poly (acrylamide/sodium methacrylate) and physically crosslinked PVA. The natural anti-freezing proteins (AFPs) inhibited the formation of ice crystals by forming hydrogen bonds with the polymer chains. The obtained hydrogel was found to perform well at −15 °C and exhibited negligible hysteresis behavior and impressive cycling durability (500 cycles at 10% strain). This design strategy could be the basis for the construction of anti-freezing protein-based conductive hydrogels.

In this section, multiple strategies to produce ETCHs are reviewed. Through the synergistic effects of ionic interactions, hydrogen bonding, surface modification, etc., the resistance to freezing and dehydration of conductive hydrogels can be further enhanced. These approaches overcome some drawbacks of the abovementioned single-method strategies for preparing ETCHs, addressing the imbalance between the mechanical properties and conductivity of hydrogels under extreme conditions and ensuring their long-term stability and durability, which is of profound significance for the further development of flexible conductive materials.

### 2.2. ETCHs with Anti-Swelling Ability 

Owing to the presence of hydrophilic groups, conductive hydrogels tend to absorb large amounts of water and experience noticeable volume expansion in aquatic environments. Excessive volume expansion negatively affects their mechanical performance, electrical conductivity, and other functions, which significantly limits their application scope. Therefore, developing conductive hydrogels with improved swelling resistance is crucial to increasing their application range in underwater environments. In this section, we report on current research developments in anti-swelling conductive hydrogels.

#### 2.2.1. Physical Interaction Mechanism

**Figure 6:**
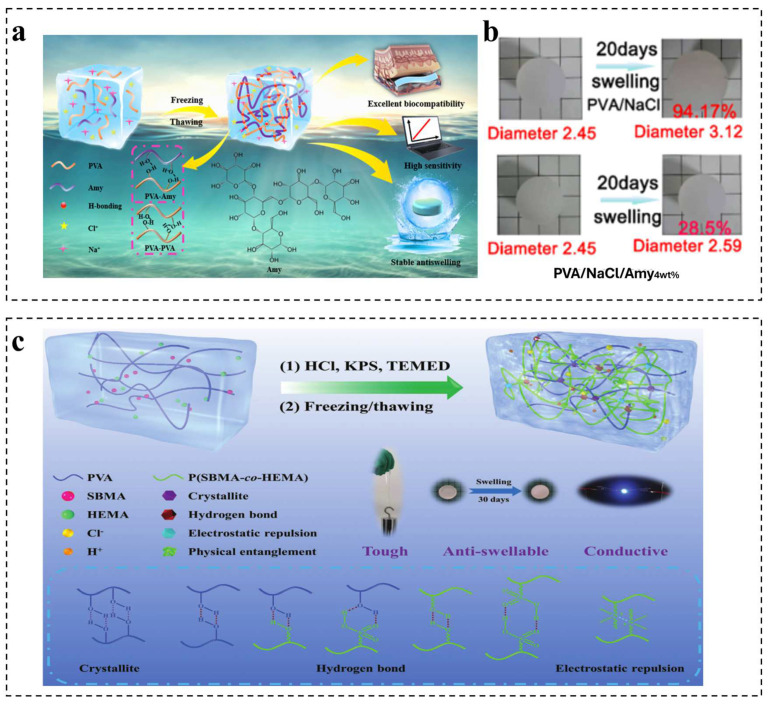
(**a**) The preparation process of anti-swelling conductive PVA/AMY hydrogel. (**b**) Images of swelling property of PVA/AMY hydrogel after swelling 20 days. From ref. [72], used with permission by Elsevier. (**c**) The synthesis process of PVA/Poly(SBMA-HEAA) anti-swelling conductive hydrogel. From ref. [73], used with permission by John Wiley and Sons.

The anti-swelling property in hydrogels can be achieved by inducing various non-covalent physical interactions, such as hydrogen bonding, electrostatic interactions, metal coordination, hydrophobic interactions, and π-π stacking [70,71]. For example, Dai’s group [72] produced an anti-swelling conductive hydrogel by employing a multi-step process, which included first adding hyper-branched AMY to PVA, then applying the freeze–thaw method, and finally immersing the hydrogel in a NaCl solution (salting-out process) (Figure 6a); as a result, PVA and AMY formed a dense network induced by strong hydrogen bonding and facilitated by salting-out effects. The hydrogel exhibited a very low swelling rate (28.5%) in aquatic environments (Figure 6b) and satisfactory toughness (184 kJ/m^3^). Lü et al. [73] developed a high-toughness, anti-swelling, and ion-conductive hydrogel comprising PVA and copolymer poly(SBMA-2-hydroxyethyl methacrylate (HEMA)), where SBMA-HEMA was polymerized within a PVA solution (Figure 6c). The electrostatic effect of the zwitterionic SBMA polymer facilitated the removal of water molecules, endowing the hydrogel with anti-swelling characteristics. After the hydrogel had been placed in water and seawater for 30 days, its swelling ratios were 9% and 13%, respectively. Additionally, the hydrogel retained 45% of its initial toughness after having been immersed in seawater for one week. Li et al. [74] proposed an innovative method to design an ETCH with anti-swelling and anti-freezing properties that involves adding macromolecular lignin to the hydrogel network. Alkali lignin (AL) was modified with methacryloyl chloride through an esterification reaction; the resulting compound was made to interact with vinyl acetate and 1-vinyl-3-butylimidazolium (ionic liquid monomers (ILs)) to form a hydrogel network. Due to the alkyl segment in ILs, the aromatic structure of AL, and the hydrogen bonding between glycerol and water, the hydrogel possessed anti-freezing and anti-swelling properties, which allowed it to withstand even the sub-zero temperature of −20 °C while maintaining a conductivity value of 10 S/m. In addition, the hydrogel was found to have satisfactory mechanical performance with elongation at a break > 350% and a tensile strength value > 1.5 MPa.

In Table 5, the anti-swelling mechanism, raw materials, properties, and applications of anti-swelling conductive hydrogels are summarized. It can be seen that the majority of anti-swelling conductive hydrogels have been applied in underwater motion sensing. However, anti-swelling conductive hydrogels obtained by inducing physical interactions might demonstrate instability due to the weak nature of such interactions. Further research needs to be performed in this field to achieve better stability in this type of hydrogel for application in aquatic environments.

#### 2.2.2. Introduction of Hydrophobic Components Mechanism

**Figure 7:**
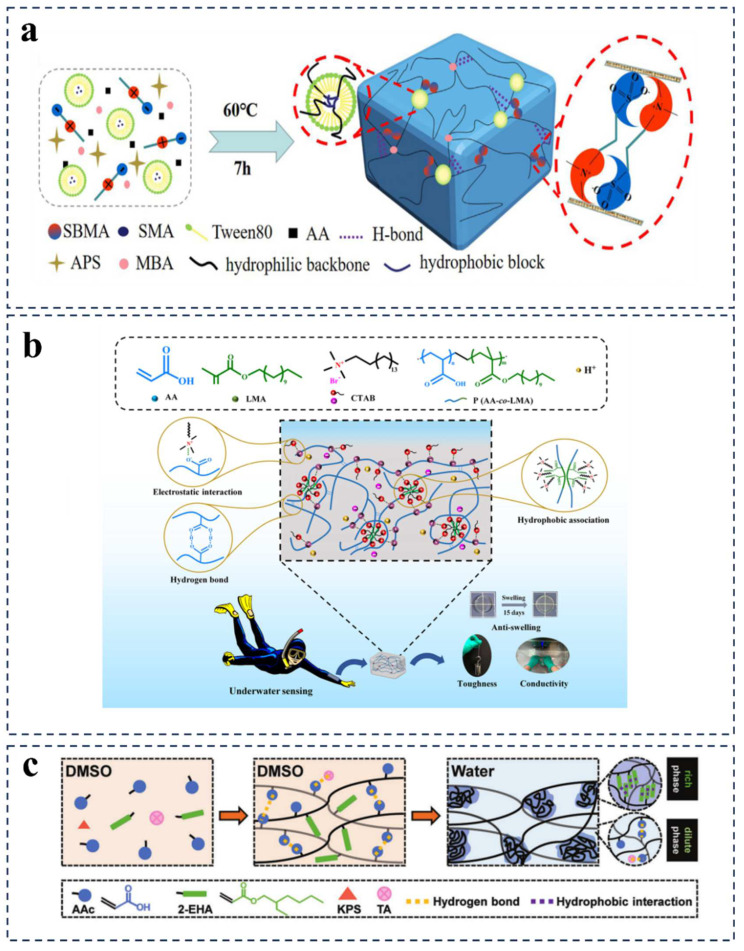
(**a**) The preparation process of AA/SMA/SBMA anti-swelling hydrogel. From ref. [78], used with permission by Royal Society of Chemistry. (**b**) The preparation process of P(AA-co-LMA)_CTAB_ low swelling conductive hydrogel. From ref. [79], used with permission by American Chemical Society. (**c**) The preparation process of AA/EHA/TA/MWCNT anti-swelling conductive hydrogel. From ref. [80], used with permission by John Wiley and Sons.

In addition to the induction of physical interactions, adding hydrophobic components to the hydrogel network is another method to achieve anti-swelling behavior in conductive hydrogels. Due to the hydrophilic components within the network, in these hydrogels, water molecules are easily exchanged and diffused, resulting in substantial volumetric expansion with the absorption of significant quantities of water in aquatic environments [75,76,77]. Conversely, when hydrophobic components are added to the hydrogel network, the diffusion of water molecules can be effectively reduced, inhibiting the excessive expansion of the hydrogel caused by water absorption. In Table 6, the raw materials, anti-swelling mechanism, anti-swelling property, and applications of anti-swelling conductive hydrogels based on hydrophobic interactions are listed and summarized. Notably, Xu et al. [78] reported an anti-swelling conductive hydrogel based on AA, octadecyl methacrylate (SMA), and SBMA (Figure 7a). The electrostatic interactions between the carboxyl groups of AA and the cations of zwitterionic SBMA, as well as the stacking of hydrophobic alkyl chains in SMA, decreased the permeability of the hydrogel, and experiments showed a swelling rate of 60% after 30 days. Additionally, the zwitterionic interaction within the hydrogel also facilitates the ion conductivity of the final anti-swelling conductive hydrogel. Dong et al. [79] developed a novel strategy for enhancing the anti-swelling property of hydrogels based on electrostatic interactions and hydrophobic interactions and produced a swelling-resistant and high-toughness supramolecular conductive hydrogel based on an ionic surfactant, cetyltrimethylammonium bromide (CTAB); a hydrophobic compound, lauryl methacrylate (LMA), with a long alkane chain; and hydrophilic AA. As shown in Figure 7b, the synergy of the electrostatic and hydrophobic interactions between CTAB and the P(AA-co-LMA) copolymer provides the hydrogel with excellent swelling resistance. The target conductive hydrogel exhibited little change in the swelling rate (~4%) after it had soaked in deionized water for 15 days. Furthermore, the P(AA-co-LMA)_CTAB_ hydrogel also exhibited excellent superior tensile strength (≈1.6 MPa) and high stretchability (>900%). Ran et al. [80] proposed a novel strategy for obtaining an anti-swelling conductive hydrogel for underwater soft, flexible bioelectronics, which they prepared by using hydrophilic/hydrophobic monomers, as shown in Figure 7c. Firstly, AA, 2-ethylhexyl acrylate (EHA), and DMSO were employed to construct the initial network structure. Then, tannic acid (TA) and a small amount of carboxylic multi-walled carbon nanotubes (MWCNTs-COOH) were added to the hydrogel network to improve the conductivity and adhesion of the target product. The designed hydrogel demonstrated remarkable stretchability (1000%) and a low swelling ratio (~40%) in aquatic environments. 

By adding hydrophobic monomers to the hydrogel matrix, the hydrophilicity of the hydrogel can be effectively reduced, thereby inhibiting excessive expansion underwater while maintaining good mechanical properties. However, hydrophobic monomers might not be soluble in aqueous solvents or be compatible with hydrophilic components. Therefore, a surfactant is usually added to the system to promote their dissolution and reactions. In addition, hydrophobic components are usually not conductive and might have a negative effect on the conductivity of the final ETCHs.

#### 2.2.3. Multiple Crosslinking Mechanism

**Figure 8:**
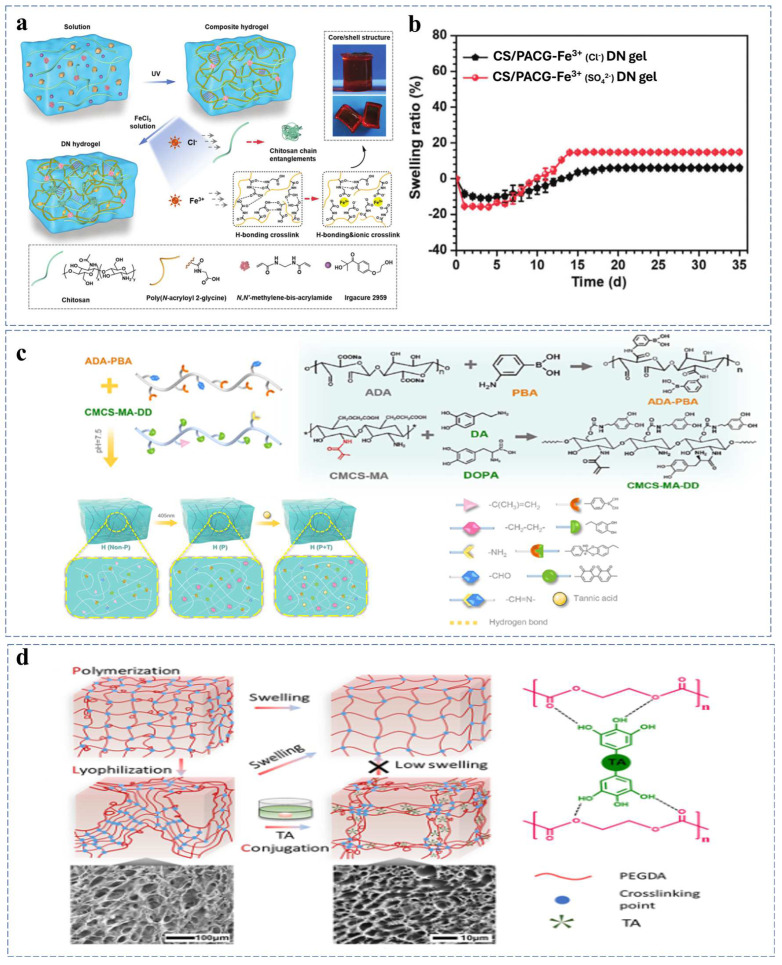
(**a**) The preparation process of CS/PACG anti-swelling conductive hydrogel. (**b**) Swelling ratio curve of CS/PACG hydrogel under different soaking solutions. From ref. [86], used with permission by John Wiley and Sons. (**c**) The preparation process of multifunctional anti-swelling conductive hydrogel. From ref. [87], used with permission by Elsevier. (**d**) The preparation process of strong and tough anti-swelling conductive hydrogel. From ref. [88], used with permission by American Chemical Society.

The molecular chains within the internal hydrogel network are influenced by the degree of crosslinking density. To achieve an anti-swelling hydrogel in line with the swelling equilibrium mechanism, it is effective in inhibiting the extension of the molecular chains by increasing the crosslinking density. However, doing so in a physically or chemically single-crosslinked conductive hydrogel may lead to defects such as fragile mechanical properties and undesirable electrical conductivity. As illustrated in Table 7, studies have shown that the mechanical properties of conductive hydrogels can be improved by inducing chemical and physical multiple crosslinking while maintaining good anti-swelling performance [82,83,84,85]. For example, Wang et al. [86] prepared a double-network composite conductive hydrogel with anti-swelling characteristics, as displayed in Figure 8a,b. First, chitosan/polyacryloyl-2-aminoethane sulfonic acid (CS/PACG) was synthesized by using UV-light-initiated polymerization. Second, the rigid/flexible dual network was obtained by adding CS/PAG to the FeCl_3_ solution for soaking, inducing hydrogen bonding and ionic interactions between Fe^3+^ and CS. The resulting hydrogel exhibited a minimum swelling ratio of 5.8% and an enhanced compressive modulus of 6.28 MPa after exposure to water for 30 days. Additionally, the swelling behavior of the hydrogel could be tuned by using different metal ion soaking solutions due to the diversity of metal coordination interactions, providing a new method for obtaining high-strength and high-toughness anti-swelling conductive hydrogels. Xie’s group [87] provided a method for the development of a new generation of multifunctional, anti-swelling conductive hydrogels, named H(P+T), based on modified carboxymethyl chitosan (CMCS) and tannic acid (TA), as shown in Figure 8c. The H(P+T) hydrogel was obtained by generating photo-polymerization-induced covalent bonding, the dynamic covalent bonding of boronic acid ester bonds, and hydrogen bonding. As a result, the final multi-crosslinked conductive hydrogel showed good anti-swelling performance with a swelling ratio of 32.4% after 30 h. It was observed that the H(P+T) hydrogel revealed a porous morphology and the smallest pore size compared with other hydrogels. Wang et al. [88] developed a high-strength and high-toughness anti-swelling hydrogel composed of polyethylene glycol diacrylate (PEGDA), quaternized chitosan (QCS), and TA, as presented in Figure 8d. First, a swelling hydrogel was prepared with PEGDA and QCS through free-radical polymerization and Michael addition. Then, the lyophilized hydrogel was immersed in TA solution for effective conjugation to obtain the final anti-swelling conductive hydrogel. According to the report, the hydrogel patch exhibited a low swelling ratio of 20.9% after five days, maintaining adhesiveness in both dry and wet environments, and making it suitable for applications requiring adhesive anti-swelling conductive hydrogels.

Physical and chemical multiple-crosslinking improves the crosslinking density and swelling resistance of conductive hydrogels, resulting in these materials maintaining stable structure and function in aquatic environments. However, in complex environments, such as acid, alkali, and salt solutions, the swelling resistance and long-term stability of conductive hydrogels remain to be studied. In addition, conductive hydrogels obtained by applying the multiple-crosslinking method might demonstrate tunable mechanical strength, as the latter can indeed be modulated by adjusting the crosslinking density; this facilitates their application in human motion or activity detection. In conclusion, the development of multiple-crosslinking methodologies has had a significant impact on the research on ETCHs.

## 3. Applications of ETCHs

Anti-freezing, anti-swelling, and anti-drying hydrogels can withstand various environmental conditions, so they have demonstrated great potential in human motion sensing, bioelectrodes, soft actuators and robots, and wound dressings. In this regard, here, we focus on the potential applications of these conductive hydrogels based on their unique characteristics.

### 3.1. Strain Sensing and Human Motion Detection

**Figure 9:**
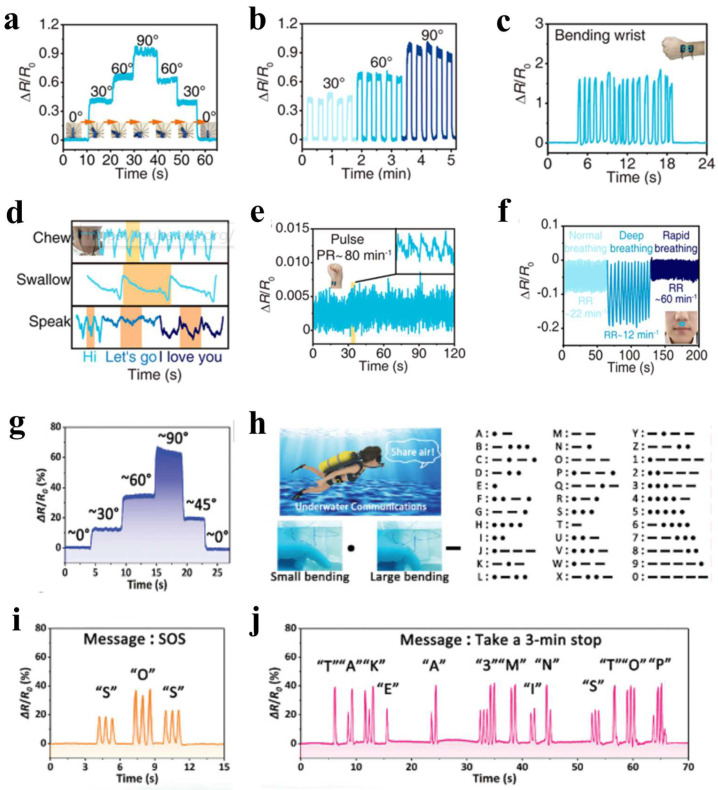
(**a**,**b**) Resistance variation of PCH-Li-G organohydrogel applied to joint flexure. (**c**) Wrist signal monitoring of PCH-Li-G organohydrogel. (**d**) Monitoring different movements of throat by PCH-Li-G organohydrogel. (**e**) Detecting the wrist pulse beats by PCH-Li-G organohydrogel. (**f**) Recording different types of breathing by PCH-Li-G organohydrogel. From ref. [93], used with permission by John Wiley and Sons. (**g**) The resistance variation of hydrophobic ionic conductive hydrogel recording different bending angles of a finger. (**h**) Schematic diagram of underwater communication mechanism by Morse code. (**i**,**j**) Sending the message “SOS” and “Take a 3-min stop” by the anti-swelling conductive hydrogel. From ref. [75], used with permission by Royal Society of Chemistry.

A flexible conductive sensing material can detect an external stimulus and convert it into an electrical signal for output. A traditional flexible sensing material composed of rigid conductive materials and flexible substrates might present the disadvantages of a complicated preparation process, insufficient elongation, and poor biocompatibility, hindering human–machine interactions, health monitoring, and motion detection. Recently, it has been found that these shortcomings can be overcome by employing ETCHs as novel sensing materials [89,90,91]. For example, Wang’s group [92] developed an organohydrogel with anti-dehydration, anti-freezing, and anti-oxidant properties composed of PVA and grape seed extract (GSP), as well as a binary solvent of water and DMSO. The obtained conductive organohydrogel did not freeze at sub-zero temperatures, not even at −23 °C. After storage at room temperature for 12 days, the organohydrogel retained 76.33% of its mass and exhibited an electrical conductivity value of 0.78 mS/cm. Therefore, in the above study, the authors demonstrated that the prepared long-lasting, environmentally resistant organohydrogel could be used as a sensor for detecting and identifying human activities at low temperatures and under UV light. Yang et al. [93] designed a double-network organohydrogel, named PCH-Li-G, composed of hyaluronic acid (HA), PAA-co-PAM, LiCl, and glycerol/water for strain/stress, humidity, and human activity sensing. The hydrogel exhibited advanced freezing resistance, with an anti-freezing temperature of −30 °C, and anti-drying performance under arid and heat conditions. The assembled PCH-Li-G hydrogel could accurately detect finger, wrist, throat, pulse, and breathing activity (Figure 9a–f). Furthermore, since humidity near the nose changes during exhalation, the sensor could distinguish among different types of human breathing. The PCH-Li3-G sensor is capable of showing a sensitive response to changes in temperature, humidity, and other factors, indicating that it maintains high sensitivity in different environments. Wei et al. [75] presented a new design for wearable sensors in underwater environments: a hydrophobic, anti-swelling ion-conductive hydrogel sensor. The sensor was prepared through heat-initiated radical polymerization, with the hydrophobic tert-butyl group acting as a barrier against water molecule penetration. Stimuli-responsive, conductive, and anti-swelling properties make the assembled sensor suitable for human motion signal monitoring (Figure 9g) and underwater communication (Figure 9h–j). Even under 400% strain, it still demonstrated a satisfactory gauge factor of 1.06. Additionally, it exhibited fast response time during stretching (37 ms) and recovery processes (98 ms). This work offered a new design conception for wearable sensors in underwater environments. Cao et al. [94] proposed a simple idea for constructing a conductive hydrogel for human–machine interaction. They first obtained a high-strength hydrogel by using a simple freeze–thaw process based on MXene, PVA solution, and cellulose scaffold from wood. The hydrogel was then immersed in glycerol to partially replace the water in the hydrogel matrix to achieve anti-freezing and anti-drying properties. The final organohydrogel could be used as an underwater intelligent sensing system for human motion detection, information communication, and target identification. Inspired by the human neuron system, Fei et al. [95] prepared a self-powered organohydrogel with anti-drying properties using gelatin, metal–organic frameworks (MOFs) modified with ionic liquids, and the binary solvent of water and glycerol. This organohydrogel could be used to monitor human motion, pressure, and humidity.

The above reports demonstrated that flexible sensors based on ETCHs possess excellent environmental adaptability, making them suitable for sensing applications in a wide temperature range, as well as arid and aqueous conditions. However, using salts or organic solvents might introduce toxic components into the hydrogel matrix, causing undesirable biocompatibility. Therefore, it is crucial to choose biocompatible anti-drying, anti-freezing, and anti-swelling chemicals for developing human motion detection sensors.

### 3.2. Bioelectrodes

**Figure 10:**
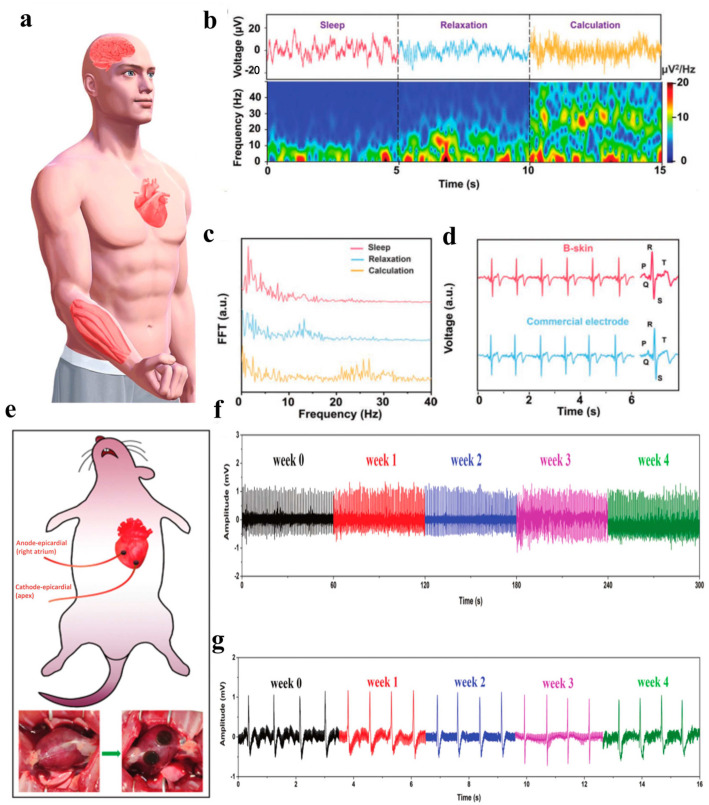
Physiological signals recording based on bioelectrodes. (**a**) Schematic diagram of the process of electronic skin attached to different parts of the human body for recording physiological signals. (**b**) Recording of EEG signals under different states. (**c**) Frequency spectrum of EEG signals processed by FFT. (**d**) Comparison of ECG signals acquired by the B-skin and commercial gel electrode. From ref. [99], used with permission by John Wiley and Sons. (**e**) Schematic illustrations of recording epicardial ECG of rats. (**f**) The epicardial ECG signals recorded by hydrogel electrodes. (**g**) The partial enlargement pictures of epicardial ECG signals. From ref. [100], used with permission by John Wiley and Sons.

Bioelectrodes can measure electrical signals in living organisms, including those from the heart (electrocardiogram (ECG) signals), the brain (electroencephalogram (EEG) signals), and muscles (electromyogram (EMG) signals), which play a significant role in human health monitoring, diagnostics, and therapeutics. Bioelectrodes are designed to have good electrical conductivity and biocompatibility to ensure accurate signal detection without causing harm to the biological tissue. ETCHs designed as bioelectrodes have been widely reported in recent years on account of their merits [96,97,98]. Inspired by the human skin structure, Mao et al. [99] prepared a biocompatible hydrogel consisting of serum proteins and glycerol. The strong hydrogen bond between the amino acids of the proteins and the hydroxyl groups of glycerol/water provides the hydrogel with anti-freezing and water-retention properties. As shown in Figure 10a–c, the hydrogel was used to detect human brain physiological signals, and the corresponding EEGs were recorded in sleep, relaxation, and calculation states. It can be seen that the signal strength and frequency changed with the brain signals in different states. By using fast Fourier transform (FFT) to analyze these brain signals, the authors revealed that higher frequencies were recorded during cognitive processing tasks, while lower frequencies were observed during periods of relaxation and sleep. Interestingly, as shown in Figure 10d, the hydrogel also accurately monitored ECG signals and showed P-QRS-T peaks. Compared with commercial electrodes, the signal-to-noise ratio of the prepared hydrogel decreased to a lesser extent after one week of utilization, indicating the long-term durability of this hydrogel in EEG monitoring. In addition, there was no significant difference in the EMG signal waveforms at the temperatures of 0 °C and 20 °C for the reported hydrogel, which also confirmed its desirable environmental stability. In another work, Zhang et al. [100] designed an anti-swelling conductive hydrogel using poly (Cu-arylacetylide) and PNIPAM. The accumulation of Cu-arylacetylide within the network enhanced the anti-swelling property of the final hydrogel. Additionally, poly (Cu-arylacetylide) also provided the hydrogel with satisfactory conductivity and anti-bacterial performance. These advantages make this hydrogel a promising material for recording various bioelectrical signals. As shown in Figure 10e, the hydrogel was used as an adhesive bioelectrode to record the epicardial ECGs of rat hearts. According to the epicardial ECG signal diagram in Figure 10f,g, the poly (Cu-arylacetylide) hydrogel electrode showed stable epicardial ECGs without obvious undulation or high-amplitude noise. Moreover, no arrhythmia signals were detected by the hydrogel electrode, indicating its excellent compatibility and adhesion when used in active tissues. Taking all the results of the above work together, the designed hydrogel represents a significant advancement in novel implantable bioelectrodes engineered using ETCHs.

Similar to human motion detection applications, in practical applications of bioelectrodes, ETCHs exhibit promising characteristics, such as high sensitivity, good mechanical performance, and environmental tolerance. These characteristics allow ETCHs not only to withstand the stress induced by human body movement and response to minor electrical stimulus signals but also to record electrical signals even in cold, aquatic, and other extreme environments. However, there are some limitations of ETCHs that still need to be addressed. For instance, fluctuations and movement relative to the skin surface (especially the occurrence of sweaty skin) may cause ETCHs to detach themselves from the skin and fall off. Therefore, it is essential to design ETCHs with enhanced adhesive performance. Moreover, the biocompatibility of ETCHs is also a crucial factor for bioelectronics applications because they interact with the skin directly.

### 3.3. Soft Actuator and Robotics

**Figure 11:**
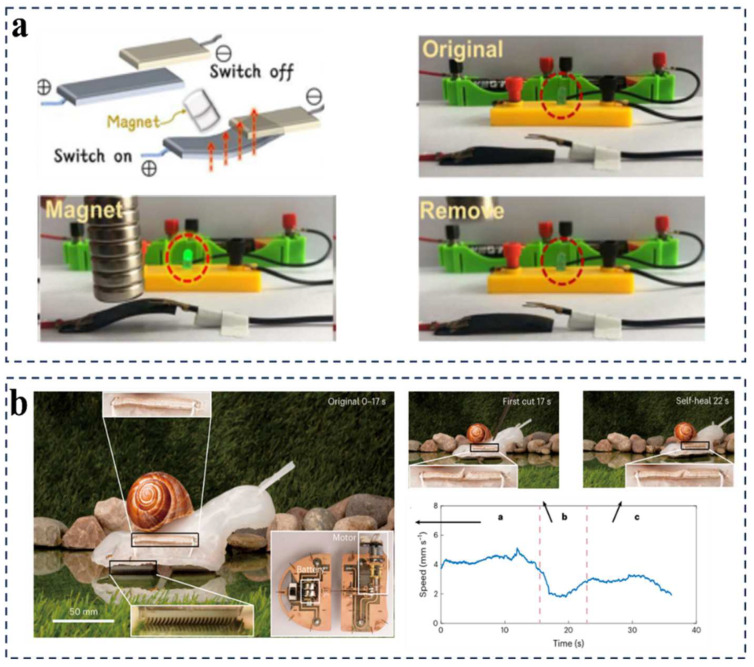
(**a**) Photography of an electrical switch controlled by a magnet. From ref. [104], used with permission by Royal Society of Chemistry. (**b**) The speed of crawling soft robot at different states. From ref. [105], used with permission by Springer Nature.

Fast technological development has led to a growing interest in intelligent soft actuators and robots that can respond to external stimuli and change their shapes/deform accordingly. Conductive hydrogels can also be employed as soft actuators and robots for tasks such as grasping delicate objects or interacting with humans because of their great stimuli-responsive characteristics and flexibility [101,102]. For example, Liu’s group [103] introduced a new organohydrogel prototype for motion sensing in soft bionic robots. They prepared an anti-freezing and anti-drying ionic organohydrogel composed of alginate and PAA, based on glycerol and NaCl solution replacement. The obtained organohydrogel displayed a satisfactory electrical conductivity value of 1.96 × 10^−5^ S/m at −70 °C, demonstrating its suitability for applications in soft robotics under sub-zero conditions. The organohydrogel was applied to the limbs of a soft robot to detect the robot’s movements in snow environments at −5.5 °C. Liu et al. [104] prepared a stretchable, robust, and anti-freezing conductive hydrogel by incorporating Fe_3_O_4_/tannic acid (TA) nanoparticles and conductive polyaniline into a phenol-carbamate/polyethylene glycol (PEG) network through in situ polymerization; then, the nanocomposite hydrogel was further soaked in glycerol/CaCl_2_ solution. The obtained hydrogel exhibited good mechanical performance (0.83 MPa tensile strength), conductivity (0.36 mS/cm), and anti-freezing properties (−20 °C). Thanks to these advantages, the ETCH was used as a bulb switch for a magnetic responsive actuator. As shown in Figure 11a, the hydrogel actuator was prepared to control the “ON/OFF” state of a bulb by virtue of its magnetic responsiveness; specifically, the hydrogel, under the influence of a magnetic field created by a magnet, could close the circuit, lighting up the bulb. After removing the magnet, the hydrogel recovered its original state, and the bulb was simultaneously turned off. Majidi et al. [105] developed an anti-drying, self-healing conductive organohydrogel using PVA, sodium borate, silver flakes, liquid metal gallium, and an organic solvent (ethylene glycol). To showcase the potential of this composite organohydrogel in soft robot applications, the researchers recorded the speed of a crawling soft robot before subjecting it to damage and during reconnection (Figure 11b).

Traditional rigid actuators and robots are limited to operating in regularly shaped free space due to their shape constraints. In contrast, soft actuators and robots based on ETCHs have the ability to undergo various deformations and exhibit better shape-changing abilities. This advantage allows them to perform tasks in irregularly shaped or limited space in extreme environments, which rigid actuators and robots are unable to accomplish. Additionally, soft actuators and robots based on ETCHs possess properties similar to those of biological tissues or organs and can be even used as substitutes for certain organs. Although extensive work has been devoted to validating the feasibility of soft actuators and robots based on ETCHs, their actuating accuracy, response period, and ability to distinguish and determine external stimulus signals are to be further investigated and improved, as it is still challenging to practically apply soft actuators and robots in everyday life.

### 3.4. Wound Dressings

The primary obstacles to wound healing encompass infection, oxidation, and inflammation. Conductive hydrogels are widely used in tissue engineering as wound dressings to support cell growth and tissue regeneration, as they can be designed to have various biological functions (e.g., anti-microbial function, anti-oxidation, anti-inflammation, wound condition monitoring, etc.), promoting wound healing. As the exposure of the wound-hydrogel system to cold, dry, and/or aqueous environments might have a negative impact on skin tissue repair, network structure, and the performance of the hydrogel [106], ETCHs with biological functions have been elaborately engineered to protect the wound from extreme environments and simultaneously promote wound healing [21,107,108]. For example, Ma et al. [109] reported an anti-freezing and anti-fouling conductive hydrogel consisting of SA, zwitterionic DMAPS monomer, and glycerol/water. First, the conductive hydrogel was prepared through the polymerization of DMAPS in SA solution and then placed into a glycerol/water solvent for replacement to manufacture the final hydrogel, named H-G_60_.

**Figure 12:**
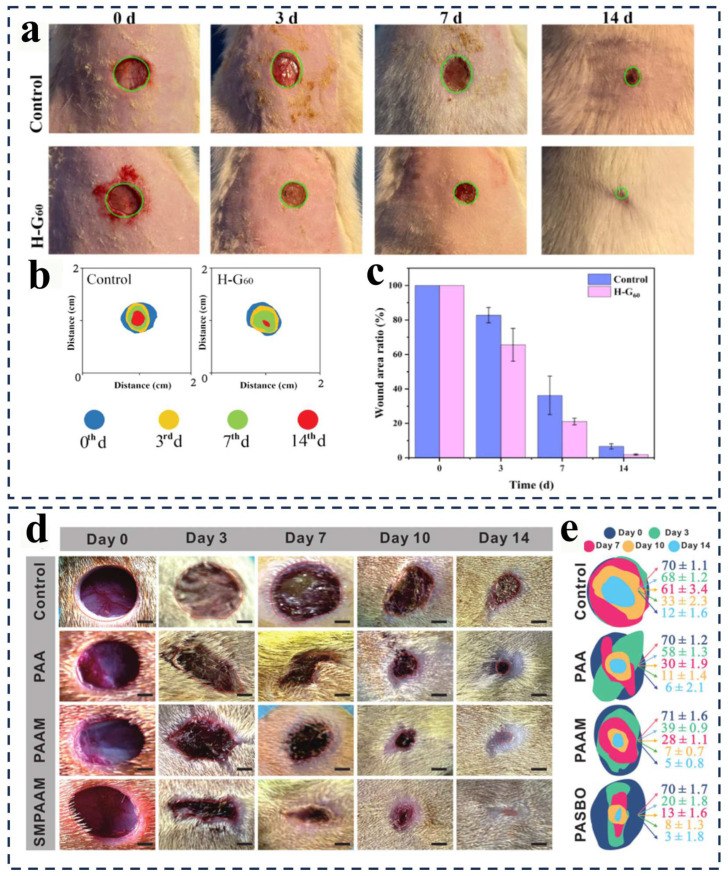
(**a**) The images of wound healing on 0, 3, 7, and 14 days. (**b**) The wound area at different times. (**c**) The statistics of wound healing areas at different times. From ref. [109], used with permission by American Chemical Society. (**d**) The shape of the wounds in each group from 0 to 14 days. (**e**) The wound area at different periods. From ref. [110], used with permission by John Wiley and Sons.

The performance of the hydrogel in wound healing was further evaluated, and the results are displayed in Figure 12a–c. It can be observed that the best performance in wound closure and subsequent healing was that of the hydrogel group after 14 days of treatment, with an ultimate wound-healing area of 97%. Wei et al. [110] designed a multifunctional anti-swelling conductive hydrogel for wound dressing by using Ti_3_C_2_ MXene and AA and a one-pot method. The engineered hydrogel was investigated for suitability for infected wound healing, as displayed in Figure 12d,e. The infected wounds tended to heal after 14 days of treatment, demonstrating the smallest wound area and the fastest wound healing in the hydrogel group. In addition, histological examination revealed that high collagen content could be found in the hydrogel group, further illustrating the wound-healing-promoting capacity of the prepared hydrogel.

Compared with regular hydrogel dressings, ETCH-based wound dressings can protect wounds in cold, dry, or aquatic environments, promoting wound healing. Additionally, these advantages can prolong the durability of ETCH-based wound dressings. However, few animal wound models have been studied in extreme environments. The wound-healing process should be researched in extreme environments to evaluate the wound-healing efficiency of ETCH-based wound dressings. Moreover, other biological functions, such as anti-bacterial, anti-inflammatory, anti-oxidant, and hemostasis functions, and angiogenesis-promoting properties, should be included in ETCH design to accelerate wound healing.

## 4. Conclusions and Outlook

In the past decade, conductive hydrogels have been extensively investigated due to their unique electrical conductivity, flexibility, viscoelasticity, biocompatibility, and stimuli-responsive properties. However, they are limited in their use in cold, dry, and/or aquatic environments due to their high water content and swelling physicochemical characteristics. To address these issues, researchers have developed ETCHs by using a variety of methodologies, such as the incorporation of salt solvents, the addition of organic solvents, surface modification with elastomer/hydrophobic layers, and regulation of the crosslinking mechanism within the hydrogel network. In this review, we summarized these strategies for preparing ETCHs with resistance to freezing, dehydration, and swelling. Further, ETCHs applied in the fields of flexible sensors, bioelectrodes, soft actuators and robots, and wound dressings were categorized and discussed. Although some progress in these fields has been made by employing ETCHs, there are still some limitations that need to be addressed for further applications, as listed below.

First, currently, the preparation process for the majority of ETCHs is complex and costly. A possible reason for this is that salt/organic solvent replacement and surface modification are more complicated than the corresponding processes in conventional conductive hydrogels. In addition, some conductive nanofillers are expensive and difficult to synthesize. Therefore, the methods for preparing ETCHs need to follow the principles of simplicity, unrestricted reaction conditions, and low cost. For example, shortening the reaction time, optimizing the cumbersome steps in the synthesis process, and selecting high-performance but low-cost raw materials could be investigated.

Second, the functionality stability of ETCHs in extreme environments remains an important challenge. Although some strategies can lower the freezing point of water to prevent ice crystal formation within the hydrogel network, the water molecules on the surface of the hydrogel inevitably freeze even at lower temperatures (such as −100 °C), which is detrimental to the hydrogel’s functionality. Moreover, the addition of organic solvents might affect the conductivity of the target ETCHs. Finally, the functionalization of hydrogels might not optimize all functional aspects. Therefore, it is crucial to balance the various properties according to the relevant application scenario. For instance, in polar or desert sensing, the design formula of ETCHs should meet extreme temperature and anti-dehydration requirements, respectively, and in underwater exploration, the anti-swelling characteristics of conductive hydrogels need to be taken into consideration. Therefore, the functionalities of ETCHs can be better regulated when their design is guided by specific application scenarios.

Third, few research studies have investigated the difference between other flexible, environmentally tolerant conductive materials or commercial products and ETCHs in the aforementioned fields. Their performance and properties should be analyzed and compared in those applications, thereby promoting the commercial production of ETCHs.

Fourth, there is little research on the utilization of ETCHs as carriers in vivo. The potential immune response of lives to ETCHs needs to be considered to ensure their safety and effectiveness. The modified hydrogels applied in vivo might trigger various types of immune responses. The design and application of modified ETCHs should be optimized to improve their biocompatibility.

In conclusion, it is expected that more novel design ideas and strategies will be designed and investigated to prepare high-strength and high-toughness hydrogels with functionalities designed for specific conditions, considering both the desired functions and the requirements of specific environments (cold, dry, and solution environments). Additionally, ETCHs could play a significant role in the development of wireless transmission technology combined with mobile terminals; existing wired transmission methods greatly limit the transmission distance of signals, but the practical application of such hydrogels underwater might improve long-distance real-time data transmission and analysis. Finally, it is anticipated that self-powering technology based on ETCHs will be developed to reduce reliance on external energy sources. Numerous studies have shown that ETCHs are among the best materials for fabricating flexible sensors, bioelectrodes, soft actuators and robots, and wound dressings. It is believed that the abovementioned limitations will be addressed and overcome, allowing ETCHs to be utilized in various extreme environments in the near future.

## Figures and Tables

**Table 1:** Performances of anti-freezing and anti-drying conductive hydrogel by immersion in salt solution strategy. “-” means not investigated.

Materials	Solvent Composition	Anti-Freezing Temperature	Anti-Drying Property	Application	Ref.
GA/PAA-CNC/betaine	CaCl_2_	−30 °C	-	Motion sensor	[35]
Cellulose/ECH	ZnCl_2_/CaCl_2_	−30 °C	-	Strain and pressure sensor	[36]
PAM/SA	LiCl	−30 °C	92% of water retention	Motion sensor	[37]
SBMA/HEAA	LiCl (6 M)	−40 °C	79.8% of water retention	Strain sensor	[38]
PVA	KAc (50 wt%)	−60 °C	90% of water retention after 10 days	Soft robot and ionic skin	[39]
SBMA/AA	LiCl (30%)	−80 °C	100% of water retention after 10 days	Human motion detection	[40]
PAM/CS/CGO	NaCl (21.2 wt%)	−56.8 °C	-	Strain sensor	[41]

**Table 3:** Performances of anti-drying and anti-swelling conductive hydrogel by surface modification. “-” means not investigated.

Materials	Structure Composition	Anti-Drying	Anti-Swelling	Application	Ref.
WPU/[EMIM][TFSI]/mHNTs	Hyrdogel/acrylic elastomer (VHB)	-	Nearly non-swelling	Underwater communication	[58]
C_18_/SDS/NaCl/MXene	Hydrogel/PDMS/Triton X-100	-	less than 2% in 30 days	Underwater communication	[59]
AA/HEMA/MXene/AgNPs	Hydrogel/Ecoflex/SiO_2_	-	No significant change	Strain sensors	[60]
Th/AM/AA/MXene	Hydrogel/PDMS	5.5% change in relative mass after 7 days	The swelling ratio less than 7%	Human motion monitoring	[61]
AM/DMA/BA/nanoclay	Hydrogel/organic gel layer	water retention rate of 95% after 2 days	3–9 wt% after 7 days	Strain sensors	[62]
AA/HEA/MXene	Hydrogel/lipogel	-	3% volume expansion in water for 200 h	Human motion detection	[63]

**Table 5:** Performance of anti-swelling conductive hydrogel induced by physical interactions. “-” means not investigated.

Materials	Interactions	Anti-Swelling Property	Application	Ref.
PVA-Gp/TA-CaCl_2_	Hydrogen bond	89% of its original weight after 30 days	Electronic skin	[70]
PVA/AMY	Hydrogen bond	28.55% of swelling ratio after 20 days	Strain sensor	[72]
PVA/poly(SBMA-HEMA)	Electrostatic interaction	9% of swelling ratio after 30 days	Underwater motion sensor	[73]
PVA-AL/ILs/H_2_O-GLY	Hydrophobic interaction and hydrogen bond	-	Human motion sensor	[74]

**Table 6:** Performance of anti-swelling conductive hydrogel induced by incorporation of hydrophobic components.

Materials	Mechanism	Swelling Ratio	Application	Ref.
t-BuA/DMAA/IL/[BMIm]TFSI	Hydrophobic/hydrophilic structure	The swelling ratio held 3.8% after 10 days	Underwater motion sensors	[75]
Liquid metal/PVA/P(AAm-co-SMA)	Hydrophobic network, ionic coordination, and hydrogen bonds	Swelling ratio less than 20% after a week	Wound dressing	[76]
AA/SMA/SBMA	Hydrophobic and hydrogen bond	1% volume swelling ratio after 30 days	Human motion sensor	[78]
AA/LMA/CTAB	Electrostatic interactions and hydrophobic associations	Around 4% of swelling ratio after 15 days	Underwater motion sensor	[79]
AA/EHA/TA/MWCNT	hydrophobic/hydrophilic structure	Swelling ratio less than 40%	Bioelectronics	[80]
AA/MEA/graphene/DMSO	Hydrophobic/hydrophilic structure	Non-swelling behavior for 500 h	Underwater motion sensors	[81]

**Table 7:** Performance of anti-swelling conductive hydrogels induced by multiple crosslinking mechanisms.

Materials	Mechanism	Swelling Ratio	Application	Ref.
AA-MEA-Fe/CS	C-C covalent bond and hydrogen bond	Around 16% after 7 days	Human motion sensor	[83]
PAA-CS-Al^3+^-MXene	C-C covalent bond, hydrogen bond, and electrostatic interactions	Swelling ratio of 3.8% after 7 days	Human motion sensor	[84]
CS/PACG	C-C covalent bond, hydrogen bond, and ionic interactions	Swelling ratio of 20% after 20 h	Wearable sensor	[86]
CMCS/SA/TA	C-C covalent bond, dynamic covalent bonds, and hydrogen bond	Swelling ratio of 32.4% for 30 h	Wound dressing	[87]
PEGDA/TA	C-C covalent bond, borate ester bond, and Schiff base bond	Swelling ratio of 20.9% after 5 days	Wound dressing	[88]

## Abbreviations

ETCHs	Environmentally tolerant conductive hydrogels
SBMA	3-[Dimethyl-[2-(2-methylprop-2-enoyloxy) ethyl]azaniumyl]propane-1-sulfonate
HEMA	2-hydroxyethyl methacrylate
PVA	Polyvinyl alcohol
AA	Acrylic acid
PAM	Polyacrylamide
CS	Chitosan
CGO	Chitosan-modified graphene oxide
CNC	Cellulose nanocrystal
GA	Arabic gum
ECH	Epichlorohydrin
SA	Sodium alginate
CNF	Cellulose nanofibers
DMSO	Dimethyl sulfoxide
TA	Tannic acid
EG	Ethylene glycol
SNF	Silk nanofibers
CN	Carbon nitride nanosheets
PDDA	Poly(diallyldimethylammonium chloride)
PA	Phytic acid
LSNs	Lignin sulfonate nanorods
MMT	Montmorillonite
G	Graphene
HEA	2-hydroxyethyl acrylate
Th	Trehalose
SDS	Sodium dodecylsulfate
C_18_	Stearylmethacrylate
BA	Butyl acrylate
DMA	N, N-dimethylacrylamide
AFP	Antifreeze proteins
AMY	Amylopectin
CMC	Carboxymethyl cellulose
VHB	acrylic elastomer
MEA	Ethylene glycol methyl ether acrylate
Gp	β-Glycerophosphate sodium
CTAB	Cetyltrimethylammonium bromide
VBIBr	1-butyl-3-vinylimidazolium bromide
[BMIm]TFSI	bis(trifluoromethanesulfonyl) imide
PEG	Poly(ethylene glycol)
AL	Alkali lignin
ILs	1-vinyl-3-butylimidazolium
MaPVA	Methacrylated polyvinyl alcohol
CMCS	Carboxymethyl chitosan
VBIPS	3-(1-(4-vinylbenzyl)-1H-benzo-[d]imidazole-3-ium-3-yl) propane-1-sulfonate
MOF	Metal–organic framework
NIR	Near infrared
ECG	Electrocardiogram signal
EEG	Electroencephalogram signal
EMG	Electromyogram signal
DMAPS	[2-(Methacryloyloxy)ethyl]dimethyl-(3-sulfopropyl)
DMAA	N,N-dimethylacrylamide
FFT	Fourier transform

## References

[1] Shi Y., Wang R., Bi S., Yang M., Liu L., Niu Z. (2023). An Anti-Freezing Hydrogel Electrolyte for Flexible Zinc-Ion Batteries Operating at −70 °C. Adv. Funct. Mater..

[2] Wu S., Lou D., Wang H., Jiang D., Fang X., Meng J., Sun X., Li J. (2022). One-pot synthesis of anti-freezing carrageenan/polyacrylamide double-network hydrogel electrolyte for low-temperature flexible supercapacitors. Chem. Eng. J..

[3] Yang J., Guo M., Feng L., Hao J., Guo Y., Li Z., Liu S., Qin G., Sun G., Chen Q. (2023). Swelling-resistant microgel-reinforced hydrogel polymer electrolytes for flexible all-in-one supercapacitors with high performances. J. Mater. Chem. C.

[4] Wang X., Weng L., Zhang X., Guan L., Li X. (2023). Constructing conductive and mechanical strength self-healing hydrogel for flexible sensor. J. Sci. Adv. Mater. Devices.

[5] Chalabianloo N., Can Y.S., Umair M., Sas C., Ersoy C. (2022). Application level performance evaluation of wearable devices for stress classification with explainable AI. Pervasive Mob. Comput..

[6] Bai H., Chen D., Zhu H., Zhang S., Wang W., Ma P., Dong W. (2022). Photo-crosslinking ionic conductive PVA-SbQ/FeCl_3_ hydrogel sensors. Colloids Surf. A.

[7] Xu Y., Gao Y., Liu R., Li J., Zhu X. (2021). Robots Built Robots: Nanorobots Customized by Intelligent Robot. Cryst. Growth Des..

[8] Chen X., Tian C., Zhang H., Xie H. (2023). Biodegradable Magnetic Hydrogel Robot with Multimodal Locomotion for Targeted Cargo Delivery. ACS Appl. Mater. Interfaces.

[9] Lee Y.W., Chun S., Son D., Hu X., Schneider M., Sitti M. (2022). A Tissue Adhesion-Controllable and Biocompatible Small-Scale Hydrogel Adhesive Robot. Adv. Mater..

[10] Wang B., Dai L., Hunter L.A., Zhang L., Yang G., Chen J., Zhang X., He Z., Ni Y. (2021). A multifunctional nanocellulose-based hydrogel for strain sensing and self-powering applications. Carbohydr. Polym..

[11] Sun X., He S., Qin Z., Li J., Yao F. (2021). Fast self-healing zwitterion nanocomposite hydrogel for underwater sensing. Compos. Commun..

[12] Han L., Zhou Q., Chen D., Qu R., Liu L., Chen Y., Yang J., Song X. (2022). Flexible sensitive hydrogel sensor with self-powered capability. Colloids Surf. A.

[13] Wang W., Deng X., Luo C. (2023). Anisotropic hydrogels with high-sensitivity and self-adhesion for wearable sensors. J. Mater. Chem. C.

[14] Moura B.S., Monteiro M.V., Ferreira L.P., Lavrador P., Gaspar V.M., Mano J.F. (2022). Advancing Tissue Decellularized Hydrogels for Engineering Human Organoids. Adv. Funct. Mater..

[15] Su G., Cao J., Zhang X., Zhang Y., Yin S., Jia L., Guo Q., Zhang X., Zhang J., Zhou T. (2020). Human-tissue-inspired anti-fatigue-fracture hydrogel for a sensitive wide-range human-machine interface. J. Mater. Chem. A.

[16] Mu G., He W., He J., Muhammad Y., Shi Z., Zhang B., Zhou L., Zhao Z., Zhao Z. (2023). High strength, anti-freezing and conductive silkworm excrement cellulose-based ionic hydrogel with physical-chemical double cross-linked for pressure sensing. Int. J. Biol. Macromol..

[17] Zhou Y., Zhang L., Lin X., Lu J., Huang Z., Sun P., Zhang Y., Xu X., Li Q., Liu H. (2023). Dual-network polyvinyl alcohol/polyacrylamide/xanthan gum ionic conductive hydrogels for flexible electronic devices. Int. J. Biol. Macromol..

[18] Peng Y., Yan B., Li Y., Lan J., Shi L., Ran R. (2020). Antifreeze and moisturizing high conductivity PEDOT/PVA hydrogels for wearable motion sensor. J. Mater. Sci..

[19] Luo J., Sun C., Chang B., Jing Y., Li K., Li Y., Zhang Q., Wang H., Hou C. (2022). MXene-Enabled Self-Adaptive Hydrogel Interface for Active Electroencephalogram Interactions. ACS Nano.

[20] Chen Y., Zhang Y., Li H., Shen J., Zhang F., He J., Lin J., Wang B., Niu S., Han Z. (2023). Bioinspired hydrogel actuator for soft robotics: Opportunity and challenges. Nano Today.

[21] Xu Z., Liu G., Huang J., Wu J. (2022). Novel Glucose-Responsive Antioxidant Hybrid Hydrogel for Enhanced Diabetic Wound Repair. ACS Appl. Mater. Interfaces.

[22] Ma Q., Li Q., Cai X., Zhou P., Wu Z., Wang B., Ma W., Fu S. (2022). Injectable hydrogels as drug delivery platform for in-situ treatment of malignant tumor. J. Drug Deliv. Sci. Technol..

[23] Yang Y., Liang Y., Chen J., Duan X., Guo B. (2022). Mussel-inspired adhesive antioxidant antibacterial hemostatic composite hydrogel wound dressing via photo-polymerization for infected skin wound healing. Bioact. Mater..

[24] Zhong Y., Seidi F., Wang Y., Zheng L., Jin Y., Xiao H. (2022). Injectable chitosan hydrogels tailored with antibacterial and antioxidant dual functions for regenerative wound healing. Carbohydr. Polym..

[25] Cheng Y., Ren X., Gao G., Duan L. (2019). High strength, anti-freezing and strain sensing carboxymethyl cellulose-based organohydrogel. Carbohydr. Polym..

[26] Yang Y., Yang Y., Cao Y., Wang X., Chen Y., Liu H., Gao Y., Wang J., Liu C., Wang W. (2021). Anti-freezing, resilient and tough hydrogels for sensitive and large-range strain and pressure sensors. Chem. Eng. J..

[27] Bei Z., Chen Y., Li S., Zhu Z., Xiong J., He R., Zhu C., Cao Y., Qian Z. (2023). A self-adhesive and low-temperature-tolerant strain sensor based on organohydrogel for extreme ice and snow motion monitoring. Chem. Eng. J..

[28] Li R., Shi Y., Alsaedi M., Wu M., Shi L., Wang P. (2018). Hybrid Hydrogel with High Water Vapor Harvesting Capacity for Deployable Solar-Driven Atmospheric Water Generator. Environ. Sci. Technol..

[29] Lei K., Chen M., Guo P., Fang J., Zhang J., Liu X., Wang W., Li Y., Hu Z., Ma Y. (2023). Environmentally Adaptive Polymer Hydrogels: Maintaining Wet-Soft Features in Extreme Conditions. Adv. Funct. Mater..

[30] Li G., Li C., Li G., Yu D., Song Z., Wang H., Liu X., Liu H., Liu W. (2022). Development of Conductive Hydrogels for Fabricating Flexible Strain Sensors. Small.

[31] Feng E., Zheng G., Li X., Zhang M., Li X., Han X., Cao L., Wu Z. (2023). A toughened, transparent, anti-freezing and solvent-resistant hydrogel towards environmentally tolerant strain sensor and soft connection. Colloids Surf. A.

[32] Xie Y., Gao S., Jian J., Shi X., Lai C., Wang C., Xu F., Chu F., Zhang D. (2023). Skin-mimicking strategy to fabricate strong and highly conductive anti-freezing cellulose-based hydrogels as strain sensors. Int. J. Biol. Macromol..

[33] Zhan Y., Fu W., Xing Y., Ma X., Chen C. (2021). Advances in versatile anti-swelling polymer hydrogels. Mater. Sci. Eng. C.

[34] Liu R., Qiao C., Liu Q., Liu L., Yao J. (2023). Fabrication and Properties of Anti-freezing Gelatin Hydrogels Based on a Deep Eutectic Solvent. ACS Appl. Polym. Mater..

[35] Xiao W., Jing F., Zhang S., Chen H., Bai L., Wang W., Yang H., Yang L., Wei D. (2023). Cellulose nanocrystal based self-healing and anti-freezing arabic gum hydrogels using betaine/CaCl_2_ anti-freeze strategy. Cellulose.

[36] Shu L., Wang Z., Zhang X.F., Yao J. (2023). Highly conductive and anti-freezing cellulose hydrogel for flexible sensors. Int. J. Biol. Macromol..

[37] Zhang C., Wang J., Li S., Zou X., Yin H., Huang Y., Dong F., Li P., Song Y. (2023). Construction and characterization of highly stretchable ionic conductive hydrogels for flexible sensors with good anti-freezing performance. Eur. Polym. J..

[38] Zhang G., Yang X., Shu H., Zhong W. (2023). Ultrahigh conductivity and antifreezing zwitterionic sulfobetaine hydrogel electrolyte for low-temperature resistance flexible supercapacitors. J. Mater. Chem. A.

[39] Wu S., Wang T.-W., Du Y., Yao B., Duan S., Yan Y., Hua M., Alsaid Y., Zhu X., He X. (2022). Tough, anti-freezing and conductive ionic hydrogels. NPG Asia Mater..

[40] Sui X., Guo H., Cai C., Li Q., Wen C., Zhang X., Wang X., Yang J., Zhang L. (2021). Ionic conductive hydrogels with long-lasting antifreezing, water retention and self-regeneration abilities. Chem. Eng. J..

[41] Li S.-N., He X.-F., Zeng Z.-F., Jiang B., Wu Q., Gong L.-X., Li Y., Bae J., Wang S., Tang L.-C. (2022). Mechanically ductile, ionically conductive and low-temperature tolerant hydrogel enabled by high-concentration saline towards flexible strain sensor. Nano Energy.

[42] Bao S., Gao J., Xu T., Li N., Chen W., Lu W. (2021). Anti-freezing and antibacterial conductive organohydrogel co-reinforced by 1D silk nanofibers and 2D graphitic carbon nitride nanosheets as flexible sensor. Chem. Eng. J..

[43] Li Z., Yin F., He W., Hang T., Li Z., Zheng J., Li X., Jiang S., Chen Y. (2023). Anti-freezing, recoverable and transparent conductive hydrogels co-reinforced by ethylene glycol as flexible sensors for human motion monitoring. Int. J. Biol. Macromol..

[44] Zhang Z., Raffa P. (2023). Anti-freezing conductive zwitterionic composite hydrogels for stable multifunctional sensors. Eur. Polym. J..

[45] Nie Y., Yue D., Xiao W., Wang W., Chen H., Bai L., Yang L., Yang H., Wei D. (2022). Anti-freezing and self-healing nanocomposite hydrogels based on poly(vinyl alcohol) for highly sensitive and durable flexible sensors. Chem. Eng. J..

[46] Li M., Chen D., Sun X., Xu Z., Yang Y., Song Y., Jiang F. (2022). An environmentally tolerant, highly stable, cellulose nanofiber-reinforced, conductive hydrogel multifunctional sensor. Carbohydr. Polym..

[47] Zong S., Lv H., Liu C., Zhu L., Duan J., Jiang J. (2023). Mussel inspired Cu-tannic autocatalytic strategy for rapid self-polymerization of conductive and adhesive hydrogel sensors with extreme environmental tolerance. Chem. Eng. J..

[48] Huang J., Wan Y., Wang M., Yang J., Sun F., Abdulkhani A., Liu X., Nawaz H., Xu F., Zhang X. (2022). Lignin nanorods reinforced nanocomposite hydrogels with UV-shielding, anti-freezing and anti-drying applications. Ind. Crop. Prod..

[49] Gao Y., Jia F., Gao G. (2022). Ultra-thin, transparent, anti-freezing organohydrogel film responded to a wide range of humidity and temperature. Chem. Eng. J..

[50] Miao C., Li P., Yu J., Xu X., Zhang F., Tong G. (2023). Dual Network Hydrogel with High Mechanical Properties, Electrical Conductivity, Water Retention and Frost Resistance, Suitable for Wearable Strain Sensors. Gels.

[51] Xia J., Luo J., Chang B., Sun C., Li K., Zhang Q., Li Y., Wang H., Hou C. (2023). High-Performance Zwitterionic Organohydrogel Fiber in Bioelectronics for Monitoring Bioinformation. Biosensors.

[52] Li Y., Liu C., Lv X., Sun S. (2021). A highly sensitive strain sensor based on a silica@polyaniline core-shell particle reinforced hydrogel with excellent flexibility, stretchability, toughness and conductivity. Soft Matter.

[53] Ding Q., Wu Z., Tao K., Wei Y., Wang W., Yang B.R., Xie X., Wu J. (2022). Environment tolerant, adaptable and stretchable organohydrogels: Preparation, optimization, and applications. Mater. Horiz..

[54] Lu Y., Yang G., Shen Y., Yang H., Xu K. (2022). Multifunctional Flexible Humidity Sensor Systems Towards Noncontact Wearable Electronics. Nano-Micro Lett..

[55] Sun H., Zhao Y., Jiao S., Wang C., Jia Y., Dai K., Zheng G., Liu C., Wan P., Shen C. (2021). Environment Tolerant Conductive Nanocomposite Organohydrogels as Flexible Strain Sensors and Power Sources for Sustainable Electronics. Adv. Funct. Mater..

[56] Dong X., Guo X., Liu Q., Zhao Y., Qi H., Zhai W. (2022). Strong and Tough Conductive Organo-Hydrogels via Freeze-Casting Assisted Solution Substitution. Adv. Funct. Mater..

[57] Qin T., Li X., Yang A., Wu M., Yu L., Zeng H., Han L. (2023). Nanomaterials-enhanced, stretchable, self-healing, temperature-tolerant and adhesive tough organohydrogels with long-term durability as flexible sensors for intelligent motion-speech recognition. Chem. Eng. J..

[58] Ma L., Wang J., He J., Yao Y., Zhu X., Peng L., Yang J., Liu X., Qu M. (2021). Ultra-sensitive, durable and stretchable ionic skins with biomimetic micronanostructures for multi-signal detection, high-precision motion monitoring, and underwater sensing. J. Mater. Chem. A.

[59] Ni Y., Zang X., Chen J., Zhu T., Yang Y., Huang J., Cai W., Lai Y. (2023). Flexible MXene-Based Hydrogel Enables Wearable Human-Computer Interaction for Intelligent Underwater Communication and Sensing Rescue. Adv. Funct. Mater..

[60] Wang Z., Zhang X., Cao T., Wang T., Sun L., Wang K., Fan X. (2021). Antiliquid-Interfering, Antibacteria, and Adhesive Wearable Strain Sensor Based on Superhydrophobic and Conductive Composite Hydrogel. ACS Appl. Mater. Interfaces.

[61] Dai S., Hu H., Zhang Y., Xu J., Zhong Y., Cheng G., Ding J. (2023). Tough hydrogel-elastomer hybrids hydrophobically regulated by an MXene for motion monitoring in harsh environments. J. Mater. Chem. C.

[62] Yang M., Wan X., Liu M., Wang Z., Jia L., Zhang F., Wang S. (2023). Wetting-Enabled Three-Dimensional Interfacial Polymerization (WET-DIP) for Bioinspired Anti-Dehydration Hydrogels. Small.

[63] Wang Z., Zhou H., Liu D., Chen X., Wang D., Dai S., Chen F., Xu B.B. (2022). A Structural Gel Composite Enabled Robust Underwater Mechanosensing Strategy with High Sensitivity. Adv. Funct. Mater..

[64] Gao Y., Gao Y., Zhang Z., Wang Y., Ren X., Jia F., Gao G. (2022). Highly conductive hydrogel sensors driven by amylose with freezing and dehydration resistances. J. Mater. Chem. C.

[65] Wang X.-y., Kim H.-J. (2022). Ultra-stretchable dual-network ionic hydrogel strain sensor with moistening and anti-freezing ability. Prog. Org. Coat..

[66] Zhou Z., Yuan W. (2023). Functionally integrated conductive organohydrogel sensor for wearable motion detection, triboelectric nanogenerator and non-contact sensing. Compos. Part A.

[67] Lu Y., Yue Y., Ding Q., Mei C., Xu X., Jiang S., He S., Wu Q., Xiao H., Han J. (2023). Environment-tolerant ionic hydrogel-elastomer hybrids with robust interfaces, high transparence, and biocompatibility for a mechanical-thermal multimode sensor. InfoMat.

[68] Wu Z., Ding Q., Li Z., Zhou Z., Luo L., Tao K., Xie X., Wu J. (2022). Ultrasensitive, stretchable, and transparent humidity sensor based on ion-conductive double-network hydrogel thin films. Sci. China Mater..

[69] Wang Y., Xia Y., Xiang P., Dai Y., Gao Y., Xu H., Yu J., Gao G., Chen K. (2022). Protein-assisted freeze-tolerant hydrogel with switchable performance toward customizable flexible sensor. Chem. Eng. J..

[70] Chen K., Liang K., Liu H., Liu R., Liu Y., Zeng S., Tian Y. (2023). Skin-Inspired Ultra-Tough Supramolecular Multifunctional Hydrogel Electronic Skin for Human-Machine Interaction. Nano-Micro Lett..

[71] Li B., Kan L., Li C., Li W., Zhang Y., Li R., Wei H., Zhang X., Ma N. (2021). Adaptable ionic liquid-containing supramolecular hydrogel with multiple sensations at subzero temperatures. J. Mater. Chem. C.

[72] Gao Y., Wang Y., Dai Y., Wang Q., Xiang P., Li Y., Gao G. (2022). Amylopectin based hydrogel strain sensor with good biocompatibility, high toughness and stable anti-swelling in multiple liquid media. Eur. Polym. J..

[73] Ren J., Liu Y., Wang Z., Chen S., Ma Y., Wei H., Lü S. (2021). An Anti-Swellable Hydrogel Strain Sensor for Underwater Motion Detection. Adv. Funct. Mater..

[74] Han X., Su Y., Che G., Zhou J., Li Y. (2023). Novel Lignin Hydrogel Sensors with Antiswelling, Antifreezing, and Anticreep Properties. ACS Sustain. Chem. Eng..

[75] Wei J., Zheng Y., Chen T. (2021). A fully hydrophobic ionogel enables highly efficient wearable underwater sensors and communicators. Mater. Horiz..

[76] Li X., Jiang M., Du Y., Ding X., Xiao C., Wang Y., Yang Y., Zhuo Y., Zheng K., Liu X. (2023). Self-healing liquid metal hydrogel for human-computer interaction and infrared camouflage. Mater. Horiz..

[77] Pi M., Qin S., Wen S., Wang Z., Wang X., Li M., Lu H., Meng Q., Cui W., Ran R. (2022). Rapid Gelation of Tough and Anti-Swelling Hydrogels under Mild Conditions for Underwater Communication. Adv. Funct. Mater..

[78] Xu T., Zhang L., Song B., Bai X., Huang Z., Bu X., Chen T., Fu H., Guo P. (2022). High-strain sensitive zwitterionic hydrogels with swelling-resistant and controllable rehydration for sustainable wearable sensor. J. Colloid Interface Sci..

[79] Qi C., Dong Z., Huang Y., Xu J., Lei C. (2022). Tough, Anti-Swelling Supramolecular Hydrogels Mediated by Surfactant-Polymer Interactions for Underwater Sensors. ACS Appl. Mater. Interfaces.

[80] Li M., Lu H., Pi M., Zhou H., Wang Y., Yan B., Cui W., Ran R. (2023). Water-Induced Phase Separation for Anti-Swelling Hydrogel Adhesives in Underwater Soft Electronics. Adv. Sci..

[81] Liu X., Zhang Q., Gao G. (2020). Solvent-Resistant and Nonswellable Hydrogel Conductor toward Mechanical Perception in Diverse Liquid Media. ACS Nano.

[82] Wei J., Xiao P., Chen T. (2023). Water-Resistant Conductive gels toward Underwater Wearable Sensing. Adv. Mater..

[83] Zhao Z., Qin X., Cao L., Li J., Wei Y. (2022). Chitosan-enhanced nonswelling hydrogel with stable mechanical properties for long-lasting underwater sensing. Int. J. Biol. Macromol..

[84] Huang H., Shen J., Wan S., Han L., Dou G., Sun L. (2023). Wet-Adhesive Multifunctional Hydrogel with Anti-swelling and a Skin-Seamless Interface for Underwater Electrophysiological Monitoring and Communication. ACS Appl. Mater. Interfaces.

[85] Di X., Hou J., Yang M., Wu G., Sun P. (2022). A bio-inspired, ultra-tough, high-sensitivity, and anti-swelling conductive hydrogel strain sensor for motion detection and information transmission. Mater. Horiz..

[86] Dou X., Wang H., Yang F., Shen H., Wang X., Wu D. (2023). One-Step Soaking Strategy toward Anti-Swelling Hydrogels with a Stiff “Armor”. Adv. Sci..

[87] Zou C.Y., Lei X.X., Hu J.J., Jiang Y.L., Li Q.J., Song Y.T., Zhang Q.Y., Li-Ling J., Xie H.Q. (2022). Multi-crosslinking hydrogels with robust bio-adhesion and pro-coagulant activity for first-aid hemostasis and infected wound healing. Bioact. Mater..

[88] Chen K., Lin Q., Wang L., Zhuang Z., Zhang Y., Huang D., Wang H. (2021). An All-in-One Tannic Acid-Containing Hydrogel Adhesive with High Toughness, Notch Insensitivity, Self-Healability, Tailorable Topography, and Strong, Instant, and On-Demand Underwater Adhesion. ACS Appl. Mater. Interfaces.

[89] Feng E., Li X., Li J., Yan Z., Zheng G., Gao W., Li Z., Ma X., Yang Z. (2021). Stretchable, healable, adhesive, transparent, anti-drying and anti-freezing organohydrogels toward multi-functional sensors and information platforms. J. Mater. Chem. C.

[90] Lu C.Y., Qiu J.H., Zhao W., Sakai E., Zhang G.H., Nobe R., Kudo M., Komiyama T. (2021). Low-temperature adaptive conductive hydrogel based on ice structuring proteins/CaCl_2_ anti-freeze system as wearable strain and temperature sensor. Int. J. Biol. Macromol..

[91] Wang S., Zhang D., He X., Zhou J., Zhou Y., Wang X., Wang Z., Liu S., Zheng S.Y., Yang J. (2022). Anti-Swelling Zwitterionic Hydrogels as Multi-Modal Underwater Sensors and All-in-One Supercapacitors. ACS Appl. Polym. Mater..

[92] Wang Z., Huang M. (2022). From grape seed extracts to extremely stable strain sensors with freezing tolerance, drying resistance and anti-oxidation properties. Mater. Today.

[93] Cai C., Wen C., Zhao W., Tian S., Long Y., Zhang X., Sui X., Zhang L., Yang J. (2022). Environment-Resistant Organohydrogel-Based Sensor Enables Highly Sensitive Strain, Temperature, and Humidity Responses. ACS Appl. Mater. Interfaces.

[94] Wang Z., Zhou Z., Wang S., Yao X., Han X., Cao W., Pu J. (2022). An anti-freezing and strong wood-derived hydrogel for high-performance electronic skin and wearable sensing. Compos. Part B.

[95] Wu K., Cui Y., Song Y., Ma Z., Wang J., Li J., Fei T., Zhang T. (2023). Neuron-Inspired Self-Powered Hydrogels with Excellent Adhesion, Recyclability and Dual-Mode Output for Multifunctional Ionic Skin. Adv. Funct. Mater..

[96] Ying B., Liu X. (2021). Skin-like hydrogel devices for wearable sensing, soft robotics and beyond. iScience.

[97] Seo J., Oh S., Choi G., Lee H.S. (2022). Anti-dryable, anti-freezable, and self-healable conductive hydrogel for adhesive electrodes. Compos. Interfaces.

[98] Ismail A.A.M., Ghanem L.G., Akar A.A., Khedr G.E., Ramadan M., Shaheen B.S., Allam N.K. (2023). Novel self-regenerative and non-flammable high-performance hydrogel electrolytes with anti-freeze properties and intrinsic redox activity for energy storage applications. J. Mater. Chem. A.

[99] Leng Z., Zhu P., Wang X., Wang Y., Li P., Huang W., Li B., Jin R., Han N., Wu J. (2023). Sebum-Membrane-Inspired Protein-Based Bioprotonic Hydrogel for Artificial Skin and Human-Machine Merging Interface. Adv. Funct. Mater..

[100] Xia X., Liang Q., Sun X., Yu D., Huang X., Mugo S.M., Chen W., Wang D., Zhang Q. (2022). Intrinsically Electron Conductive, Antibacterial, and Anti-swelling Hydrogels as Implantable Sensors for Bioelectronics. Adv. Funct. Mater..

[101] Guo M., Yang X., Yan J., An Z., Wang L., Wu Y., Zhao C., Xiang D., Li H., Li Z. (2022). Anti-freezing, conductive and shape memory ionic glycerol-hydrogels with synchronous sensing and actuating properties for soft robotics. J. Mater. Chem. A.

[102] Lin F., Qiu Y., Zheng X., Duanmu Z., Lu Q., Huang B., Tang L., Lu B. (2022). One-pot mechanochemical assembly of lignocellulose nanofiber/graphite nanocomposites for wearable electronic devices. Chem. Eng. J..

[103] Liu Z., Wang Y., Ren Y., Jin G., Zhang C., Chen W., Yan F. (2020). Poly(ionic liquid) hydrogel-based anti-freezing ionic skin for a soft robotic gripper. Mater. Horiz..

[104] Liu Y., Zhang Z., Yang X., Li F., Liang Z., Yong Y., Dai S., Li Z. (2023). A stretchable, environmentally stable, and mechanically robust nanocomposite polyurethane organohydrogel with anti-freezing, anti-dehydration, and electromagnetic shielding properties for strain sensors and magnetic actuators. J. Mater. Chem. A.

[105] Zhao Y., Ohm Y., Liao J., Luo Y., Cheng H.-Y., Won P., Roberts P., Carneiro M.R., Islam M.F., Ahn J.H. (2023). A self-healing electrically conductive organogel composite. Nat. Electron..

[106] Jian Y., Handschuh-Wang S., Zhang J., Lu W., Zhou X., Chen T. (2021). Biomimetic anti-freezing polymeric hydrogels: Keeping soft-wet materials active in cold environments. Mater. Horiz..

[107] Teng K., An Q., Chen Y., Zhang Y., Zhao Y. (2021). Recent Development of Alginate-Based Materials and Their Versatile Functions in Biomedicine, Flexible Electronics, and Environmental Uses. ACS Biomater. Sci. Eng..

[108] Wei Q., Chen K., Zhang X., Ma G., Zhang W., Hu Z. (2022). Facile preparation of polysaccharides-based adhesive hydrogel with antibacterial and antioxidant properties for promoting wound healing. Colloids Surf. B.

[109] Tian S., Wang M., Wang X., Wang L., Yang D., Nie J., Ma G. (2022). Smart Hydrogel Sensors with Antifreezing, Antifouling Properties for Wound Healing. ACS Biomater. Sci. Eng..

[110] Wei C., Tang P., Tang Y., Liu L., Lu X., Yang K., Wang Q., Feng W., Shubhra Q.T.H., Wang Z. (2022). Sponge-Like Macroporous Hydrogel with Antibacterial and ROS Scavenging Capabilities for Diabetic Wound Regeneration. Adv. Healthc. Mater..

